# Prognostic Impact of Oncogenic Fibroblast Growth Factor Receptor Alterations in Patients With Advanced Solid Tumors in a Real‐World Setting

**DOI:** 10.1002/cam4.70546

**Published:** 2025-02-25

**Authors:** Levon Demirdjian, Spyros Triantos, Kristopher Standish, Shibu Thomas, Qi Xia, Jiarui Zhang, Joel Greshock, Julie Paone, Paige Sheridan, Shubham Pant, Christophe Massard, David A. Reardon, Yohann Loriot, Martin Schuler, Hussein Sweiti

**Affiliations:** ^1^ Janssen Research & Development Spring House Pennsylvania USA; ^2^ Aetion New York New York USA; ^3^ The University of Texas, MD Anderson Cancer Center Houston Texas USA; ^4^ Gustave Roussy Université Paris Saclay Villejuif France; ^5^ Dana‐Farber Cancer Institute and Harvard Medical School Boston Massachusetts USA; ^6^ West German Cancer Center University Hospital Essen Essen Germany

**Keywords:** erdafitinib, fibroblast growth factor inhibitor, real‐world evidence, tumor agnostic

## Abstract

**Background:**

Somatic *FGFR* gene alterations (*FGFRalt*) may act as oncogenic drivers across several cancers. The prognostic impact of *FGFRalt* in solid tumors is not fully understood. We assessed the prognostic impact of *FGFRalt* on overall survival (OS) in a tumor‐agnostic real‐world cohort of patients with advanced solid tumors.

**Methods:**

This was a retrospective, observational, comparative cohort analysis that used data from a nationwide de‐identified clinico‐genomic database. Patients were included if they had advanced/metastatic disease, were aged ≥ 18 years at the time of diagnosis, had evidence of genomic testing for *FGFRalt*, and had initiated first‐line systemic therapy for their cancer. Patients without *FGFR* alterations (*FGFRneg*) were matched 3:1 with patients with *FGFRalt* using a combination of exact matching on tumor type and Mahalanobis‐distance matching on selected clinical confounders. The primary endpoint was OS from time of initiation of first‐line therapy in patients with *FGFRalt* versus *FGFRneg*. To further mitigate bias, delayed entry models and covariate‐adjusted stratified Cox models were implemented.

**Results:**

The final cohort included 1012 patients (253 *FGFRalt*, 759 *FGFRneg*), across 30 tumor types. There were no significant differences in real‐world OS from first‐line therapy between *FGFRalt* and *FGFRneg* groups (hazard ratio 0.97; *p* = 0.78). Median OS from initiation of first‐line therapy was 1.13 years (95% confidence interval [CI] 0.92–1.52) and 1.01 years (0.89–1.15) for the *FGFRalt* and *FGFRneg* groups, respectively.

**Conclusions:**

In this matched‐cohort real‐world analysis, presence of *FGFRalt* had no impact on the prognosis of patients with advanced solid tumors receiving standard‐of‐care treatment.

## Graphical abstract

In this statistically powered and matched‐cohort real‐world analysis, there was no statistically significant difference in real‐world overall survival between matched patients with *FGFR*‐altered or *FGFR*‐negative advanced solid tumors, with a median overall survival of ~1 year in both groups. These results highlight the poor prognosis of patients with advanced solid tumors harboring *FGFR* alterations and the need for novel, targeted therapies.

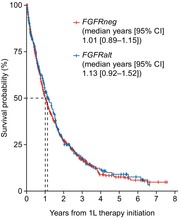

## 1 Introduction

Fibroblast growth factor receptors (FGFRs) are part of the family of tyrosine kinase receptors and are involved in cell proliferation and growth, among other biological processes [1]. Somatic *FGFR* gene alterations (*FGFRalt*), including *FGFR2* single nucleotide variants (SNVs) and *FGFR2/3* fusions, are proposed to be oncogenic drivers in many solid tumors [2, 3, 4]. Though seen most commonly in bladder cancer (20%–70% in high‐grade and low‐grade bladder cancers, respectively), *FGFRalt* occurs at various frequencies across a wide range of malignancies [2, 3, 5, 6, 7, 8, 9], including non–small‐cell lung cancer (NSCLC; *FGFR3/2*, ~3%) [6, 7] and cholangiocarcinoma (CCA; 10%–16%, *FGFR2* fusion, ~45%) [8, 9, 10]. Patients with advanced solid tumors in whom *FGFRalt* may be observed [11], in general, have poor outcomes and limited therapeutic options. For patients with many tumor histologies, median survival after diagnosis of advanced or metastatic disease is < 1 year [12, 13, 14]. For example, in patients with glioblastoma, in whom *FGFRalt* have been estimated to occur at a frequency of ~3%, treatment with approved second‐line therapies has resulted in a median survival of 9 months [12]. Although prognosis is generally poor for patients with advanced solid tumors with *FGFRalt*, the extent to which *FGFRalt* itself affects prognosis is not fully understood.

A more comprehensive understanding of the role of oncogenic driver mutations on patient survival has elucidated the potential effects of tailored approaches to treating various solid tumors. For example, NSCLC driven by *EGFR* mutations comprises approximately 15% of all NSCLC in North America; *EGFR* exon 19del or L858R substitution mutations are the most frequently reported (approximately 85% of all *EGFR*‐mutated NSCLC) [15]. These alterations are associated with increased sensitivity to EGFR tyrosine kinase inhibitors (TKIs), leading to improved prognosis and favorable survival. Similarly, in NSCLC, *STK11* mutations are associated with poor prognosis and resistance to immune checkpoint inhibitors such as PD‐1/PD‐L1 inhibitors, and have been linked to a higher likelihood of metastasis and shorter survival [16, 17]. In addition, breast cancer studies have used bioinformatics and statistical expression data that have identified key prognostic genes [18, 19] and prognostic chemokines [20]. The prognostic impact of *FGFRalt* on survival in patients with urothelial cancer (UC) and CCA has been reported in the literature, with findings showing mixed results, and the prognostic impact of *FGFRalt* in other solid tumors has not been determined comprehensively.

To date, FGFR‐targeted therapies have received approvals for histology‐specific indications (CCA and UC), but not for histology‐agnostic indications. As the common result of *FGFRalt* across tumor types may be activated FGFR canonical pathway signaling, patients with *FGFRalt*, irrespective of solid tumor type, may benefit from treatment with FGFR inhibitors. Pan‐FGFR inhibitors, such as infigratinib, futibatinib, and pemigatinib, were granted accelerated approval by the US Food and Drug Administration (FDA) for patients with previously treated, unresectable, locally advanced or metastatic CCA with an *FGFR2* fusion or other rearrangement [21, 22, 23]. Erdafitinib is a pan‐FGFR inhibitor approved by the FDA for adult patients with locally advanced or metastatic UC with susceptible *FGFR3* alterations, as determined by an FDA‐approved companion diagnostic test, whose disease has progressed on or after at least one line of prior systemic therapy, with additional approvals across geographies [24, 25, 26, 27]. RAGNAR (NCT04083976) is an ongoing phase 2, open‐label, single‐arm study to examine the safety and efficacy of erdafitinib in patients with advanced solid tumors (excluding UC) with prespecified *FGFRalt* [28]. In the RAGNAR study primary analysis, erdafitinib demonstrated an overall response rate of 30%, a disease control rate of 74%, and a median overall survival (OS) of 10.7 months. As RAGNAR is a single‐arm study with no comparator arm, an understanding of the prognostic value of *FGFRalt* across tumor types can help contextualize the observed outcomes and can facilitate an understanding of the unmet medical need in populations that do not have access to FGFR inhibitors in routine care.

We report findings of a tumor‐agnostic observational study aimed to assess the prognostic impact of *FGFRalt* (contextualized to RAGNAR with similarly defined *FGFRalt*) on real‐world OS (rwOS) of patients in a tumor‐agnostic setting.

## 2 Methods

### 2.1 Dataset

This was a protocol‐driven, retrospective, observational, comparative cohort analysis using data from the Flatiron Health (FH)‐Foundation Medicine Inc. (FMI) Clinico‐Genomic Database (CGDB). FMI directly links de‐identified comprehensive genomic profiling data and other biomarker data to de‐identified longitudinal clinical, treatment, and real‐world outcomes data from FH by de‐identified, deterministic matching [29]. Advanced/metastatic disease was defined by classifying patients into tumor‐specific databases or a pan‐tumor database (as defined in Data S1). *FGFR* test results were generated from archival tumors using the FoundationOne CDx (Cambridge, MA) targeted DNA sequencing platform, which included comprehensive genomic profiling of > 300 cancer‐related genes [30]. Notably, this includes *FGFR*1/2/3/4 loci and is designed to sensitively capture small sequence mutations and gene fusions associated with canonical pathway activation in cancer cells. The CGDB is US based and includes patients from more than 280 cancer clinics (~800 sites; primarily community setting) who have undergone genomic testing. Patients were required to have at least two visits recorded in the CGDB to ensure that they were receiving care versus consultation services (consistent with previous analyses of the dataset). The de‐identified data were subject to obligations to prevent re‐identification and protect patient confidentiality.

### 2.2 Inclusion and Exclusion Criteria

Patients (aged ≥ 18 years) were diagnosed with advanced/metastatic disease between January 1, 2011, and December 31, 2020, had evidence of comprehensive genomic testing for *FGFR* alterations, and had initiated first‐line systemic treatment for advanced/metastatic disease. Patient distribution by tumor type reflects real‐world prevalence of *FGFRalt* and *FGFR* testing by tumor type and in the CGDB. Tumor types represented in the final analysis dataset included high‐grade (grade ≥ 3) glioma, including glioblastoma, CCA, and NSCLC (full listing in Data S1). Patient exclusion criteria included diagnosis of UC (per alignment with the RAGNAR study [11]) and use of a selective FGFR inhibitor. Additional exclusion criteria are in Data S1.

### 2.3 Molecular Diagnostics

*FGFRalt* were selected to align with the RAGNAR study [11] and were defined as one or more of 81 prespecified *FGFR* oncogenic driver short sequence mutations or any *FGFR* fusion involving an intact kinase domain (Figure S1) [11]. In line with the RAGNAR study, patients with any of the following *FGFR* valine gatekeeper or resistance alterations (alterations that confer resistance to FGFR inhibition) at any time were excluded as kinase domain mutations may drive resistance and bypass tyrosine kinase inhibition [31]: *FGFR1* V561, *FGRF2* V564, *FGFR3* V555, *FGFR4* V550, *FGFR1* N546, *FGFR2* N549, *FGFR3* N540, and *FGFR4* N535. To account for multiple versions of the genomic testing platform used to determine *FGFR* status in the dataset, test version was added as a variable in the Mahalanobis‐distance matching algorithm used to match patients with *FGFRalt* and *FGFR* wild‐type (*FGFRneg*) tumors.

### 2.4 Outcome Measures

The primary endpoint was rwOS, defined as time from the start of first‐line treatment in the advanced/metastatic setting to death from any cause. Patients for whom death was not recorded were censored at the earliest time they met a prespecified censoring criterion (defined in Data S1). Briefly, patients who died without meeting any censoring criteria were counted as having a death event. Patients who died but met a censoring criterion prior to death were censored at the earliest time they met the censoring criterion. Deaths that occurred after last clinical activity but before the end of the data cut were counted as outcomes (deaths) rather than censored at last clinical activity (assuming no other censoring criteria were met). Evidence of death was based on several data sources, including external sources linked to the Social Security Death Index and obituary data (defined in the “Mortality capture” section in Data S1).

Secondary endpoints were included in an exploratory capacity and included rwOS by tumor type and line of therapy (LOT) and real‐world time to next treatment (rwTNT) across tumor types, by tumor type, and by LOT (complete list in Data S1).

### 2.5 Study Index Date Assignment

The index dates of patients were assigned as follows:

- Patients classified into one of the tumor‐specific databases: Initiation of first‐line treatment.
- Patients classified into the pan‐tumor database: First non‐canceled medication order, administration, or abstracted oral therapy after advanced/metastatic diagnosis.

**FIGURE 1:**
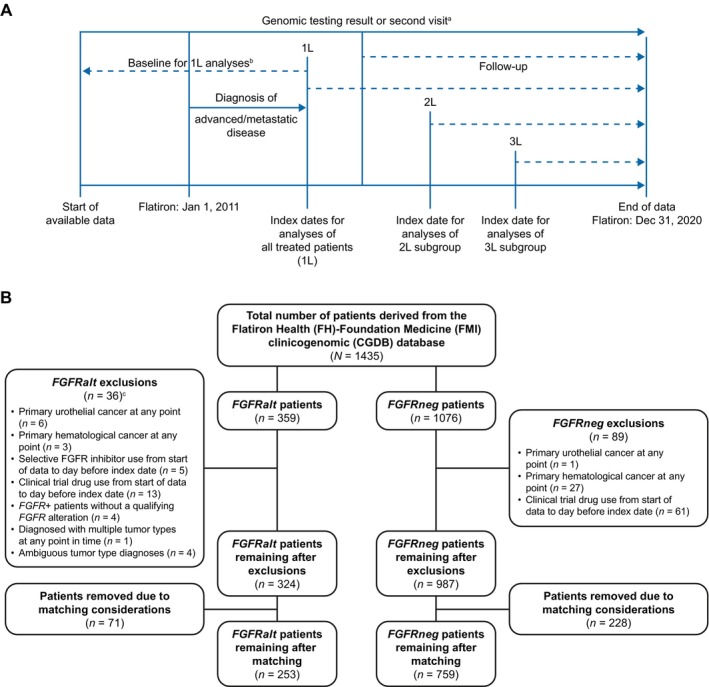
(A) Key study time periods and (B) CONSORT diagram including cohort selection criteria across all tumors. 1L, first‐line; 2L, second‐line; 3L, third‐line; *FGFRalt*, fibroblast growth factor receptor gene alterations; *FGFRneg*, fibroblast growth factor receptor gene without mutations or alterations. ^a^Date of genomic testing or date of second visit will be used as the delayed entry date if it occurred after initiation of therapy. ^b^Using all available data. ^c^Exclusion criteria were not mutually exclusive. One patient with *FGFRalt* met two exclusion criteria, resulting in 35 patients being removed.

Key time periods for the analyses are shown in Figure 1A. For analyses for which the start date is first‐line therapy initiation, the baseline period included all available data prior to first‐line therapy. For analyses of rwOS and treatment patterns among the subgroups of patients with second‐ and third‐line therapies for which the index date is second‐ or third‐line initiation, the baseline periods included all available data prior to second‐ and third‐ line initiation, respectively. There was no minimum requirement for baseline data.

Delayed entry models (adjustments for delayed entry left truncation described in Data S1) were used to mitigate immortal time bias, which could arise because patients included in the analysis needed to survive long enough to be tested for *FGFR* alterations and to have had at least two visits. During this period, the patients could not have died (hence “immortal”) [32]. Patients who died or were lost to follow‐up before genomic testing or their second visit were excluded. To address potential violations of the assumptions required for delayed entry models, non‐delayed entry and landmark sensitivity analyses were also included.

### 2.6 Statistical Analysis

*FGFRneg* and *FGFRalt* patient groups were matched 3:1 based on tumor type, age/date of advanced/metastatic diagnosis, sex, and genomic test platform version using a combination of exact (tumor type; note that disease type classifications are defined by the clinical study protocol NCT04083976) [28] and Mahalanobis‐distance (all other select matching variables) matching (list and rationale for used variable selection in Data S1). Due to exact matching by tumor type, both *FGFRalt* and *FGFRneg* groups had exactly the same distribution of tumor types.

The hazard ratio (HR) comparing rwOS for the *FGFRalt/FGFRneg* patient groups was estimated for the matched data using a Cox proportional hazards model accounting for delayed entry. A tumor‐type stratified Cox model was used for the primary analysis to account for presumed differences in baseline hazard functions for the different tumor types. An unstratified Cox model was included as a sensitivity analysis, which, together with additional sensitivity analyses, is described in Data S1. Cox models were adjusted for additional covariates of interest. In the event that there were model convergence issues due to small sample sizes (*n* < 10 patients) or a small number of events, tumor types with small sizes were grouped together to achieve *n* ≥ 10 patients, per protocol.

Prespecified covariates of interest included in the Cox models were tumor stage, tumor mutational burden (TMB), Eastern Cooperative Oncology Group performance status (ECOG PS), Charlson Comorbidity Index (CCI), smoking history, diagnosis of cancer at advanced/metastatic disease (vs prior to advanced/metastatic disease), number of prior LOTs (i.e., systemic therapies prior to the diagnosis of metastatic disease), and previous medications. Selection was based on clinical judgment (e.g., the potential to be a confounder on the causal pathway from *FGFR* status to survival), published literature, data availability, and differences in cohort distributions. Standardized mean differences (SMDs) were used to determine the balance of variables post matching, with variables achieving an absolute SMD < 0.10 deemed balanced [33, 34].

The study was statistically powered for the primary endpoint of rwOS in the tumor‐agnostic setting. Due to the rarity of qualifying target *FGFR* mutations and fusions, the availability of *FGFRalt* patient source data was used for sample size consideration, with power calculations used to select the number of matches for each patient with *FGFRalt* (Table S1). No corrections for multiple hypothesis testing were implemented.

Censoring rules are provided in Data S1.

## 3 Results

### 3.1 Patient Characteristics

Data from 1435 patients (359 *FGFRalt* and 1076 matched *FGFRneg*) were available in the CGDB (Figure 1B). After data ingestion, a subset of patients was removed from the analysis dataset, prior to analysis, because they met the exclusion criteria stated in the protocol or due to matching considerations (e.g., removing all *FGFRneg* matches to a patient with *FGFRalt* who was removed due to meeting an exclusion criteria). The final analysis set comprised 1012 patients (253 *FGFRalt*, 759 *FGFRneg*).

All variables used for matching patients with *FGFRalt* and those who are *FGFRneg* (tumor type, age/date of advanced/metastatic diagnosis, sex, and genomic test platform version) were balanced after matching, achieving a post‐matching absolute standardized difference in means/proportions < 0.10. Smoking history, time from advanced/metastatic disease diagnosis to frontline treatment initiation, ECOG PS values, and modified CCI score were balanced across groups (Table 1 [unmatched data in Table S2] and Table S3; Table S4 by mutation or fusion for *FGFRalt*). Variables that had absolute SMDs > 0.10 after matching included race (White and other categories), group stage at initial diagnosis (stages 2 and 4), TMB, and components of the CCI, including prior myocardial infarction, congestive heart failure, and mild chronic liver disease (see Table 1 and Table S3; Table S4 by mutation or fusion for the full list of variables). Covariate adjustment was used to mitigate any residual imbalances in variables after matching. The most common treatment regimens by LOT for specific tumor types are shown in Table S5. The median (interquartile range) number of LOTs that patients underwent was 2 (1‐3) in both the *FGFRalt* and *FGFRneg* groups and was balanced across tumor types. There were 30 different tumor types included in the final analysis set. CCA, NSCLC, and breast cancer were the most prevalent entities (Table 2). The most common *FGFR* fusions and mutations varied across tumor types (Table S6).

**TABLE 1:** Demographic and baseline characteristics of *FGFRalt/FGFRneg* groups across all tumors.

Characteristic	*FGFRalt* (*n* = 253)	*FGFRneg* (*n* = 759)	Absolute standardized difference in means/proportions
Demographic characteristics among all tumors			
Age at advanced/metastatic diagnosis Median (range), years	62 (18–84)	63 (28–85)	
< 65 years	147 (58.1)	426 (56.1)	0.06
≥ 65 years	106 (41.9)	333 (43.9)	0.04
Sex			
Male	113 (44.7)	339 (44.7)	0.00
Female	140 (55.3)	420 (55.3)	
Race			
White	183 (72.3)	503 (66.3)	0.13
Black or African American	21 (8.3)	53 (7.0)	0.05
Asian	4 (1.6)	18 (2.4)	0.06
Hispanic or Latino	1 (0.4)	2 (0.3)	0.02
Other	30 (11.9)	127 (16.7)	0.14
Unknown	14 (5.5)	56 (7.4)	0.08
Smoking history			
Yes No Unknown	140 (55.3) 113 (44.7) 0 (0.0)	438 (57.7) 319 (42.0) 2 (0.3)	0.05 0.05 0.07
Clinical characteristics among all tumors			
Group stage at initial diagnosis			
Group stage 0	18 (7.1)	52 (6.9)	0.01
Group stage 1	27 (10.7)	87 (11.5)	0.03
Group stage 2	51 (20.2)	109 (14.4)	0.15
Group stage 3	128 (50.6)	385 (50.7)	0.00
Group stage 4	29 (11.5)	123 (16.2)	0.14
Missing	0 (0.0)	3 (0.4)	0.14
Tumor mutational burden (version 1)^a^	43 (17.0)	152 (20.0)	
Median (IQR)	6.09 (1.25–15.66)	4.35 (1.93–8.63)	0.44
Tumor mutational burden (version 2)^b^	41 (16.2)	133 (17.5)	
Median (IQR)	7.02 (1.25–15.76)	5.04 (2.52–9.65)	0.43
Time from advanced/metastatic disease to first systemic treatment	252 (99.6)	747 (98.4)	
Median (IQR), months	31.50 (16.25–54.0)	29.0 (16.0–50.0)	0.06
Clinical characteristics at baseline			
ECOG PS			
0	81 (32.0)	238 (31.4)	0.01
1	80 (31.6)	212 (27.9)	0.08
2	19 (7.5)	50 (6.6)	0.04
3	6 (2.4)	14 (1.8)	0.04
4	0 (0)	1 (0.1)	0.05
Missing	67 (26.5)	244 (32.1)	0.13
Modified CCI score Median (IQR)	1.00 (0.0–2.0)	1.00 (0.0–2.0)	0.08

*Note:* All values are *n* (%) except where otherwise noted. Variables used in the matching algorithm were balanced between *FGFRalt*/*FGFRneg* groups in the analyses of all patients but may have become unbalanced in analyses of patient subgroups who initiated second‐ and third‐line treatment, and patient subgroups based on treatment type for specific tumor types. Imbalances were accounted for by including the variables used for matching in multivariate covariate adjustment.

Abbreviations: CCI, Charlson Comorbidity Index; ECOG PS, Eastern Cooperative Oncology Group performance status; *FGFRalt*, fibroblast growth factor receptor gene alterations; *FGFRneg*, fibroblast growth factor receptor gene without mutations or alterations; IQR, interquartile range.

^a^ Version 1 approximates the tumor mutational burden score from running all samples and is biomarker‐harmonized to incorporate recent scientific understanding.

^b^ Version 2 approximates clinically reported values and the variant annotations used are based on latest scientific understanding at the time of reporting.

**TABLE 2:** Distribution of tumor types in all patients.

Tumor type	*FGFRalt* (*n* = 253)	*FGFRneg* (*n* = 759)	Total (*N* = 1012)
Tumor‐specific databases^a^			
Cholangiocarcinoma	66 (26.1)	198 (26.1)	264 (26.1)
Non‐small cell lung cancer	45 (17.8)	135 (17.8)	180 (17.8)
Breast cancer	32 (12.6)	96 (12.6)	128 (12.6)
Gastric/esophagogastric cancer	15 (5.9)	45 (5.9)	60 (5.9)
Colorectal cancer	13 (5.1)	39 (5.1)	52 (5.1)
Melanoma	12 (4.7)	36 (4.7)	48 (4.7)
Endometrial cancer	9 (3.6)	27 (3.6)	36 (3.6)
Head and neck cancer	7 (2.8)	21 (2.8)	28 (2.8)
Pancreatic cancer	5 (2.0)	15 (2.0)	20 (2.0)
Ovarian cancer	3 (1.2)	9 (1.2)	12 (1.2)
Hepatocellular carcinoma	2 (0.8)	6 (0.8)	8 (0.8)
Small cell lung cancer	2 (0.8)	6 (0.8)	8 (0.8)
Prostate cancer	1 (0.4)	3 (0.4)	4 (0.4)
Pan‐tumor database^b^			
Glioblastoma	11 (4.3)	33 (4.3)	44 (4.3)
Carcinoma: Gyn: Cervical	10 (4.0)	30 (4.0)	40 (4.0)
Other^c^	20 (7.9)	60 (7.9)	80 (7.9)

*Note:* All values are *n* (%).

Abbreviations: *FGFRalt*, fibroblast growth factor receptor gene alterations; *FGFRneg*, fibroblast growth factor receptor gene without mutations or alterations.

^a^ The Clinico‐Genomic Database includes patients with both a confirmed diagnosis of advanced or metastatic cancer and an available *FGFRalt* status. Additional criteria are required for a patient to qualify for a tumor‐specific database. Tumor‐specific databases include those shown plus renal cell carcinoma.

^b^ If the patient is not eligible for any tumor‐specific database, the patient becomes eligible for the pan‐tumor database. With this hierarchy, a patient cannot exist in both a tumor‐specific database and the pan‐tumor database.

^c^ Other includes (*FGFRalt*/*FGFRneg*; *n*): Cervical carcinoma (10/30); anal carcinoma (3/9); vaginal carcinoma (3/9); carcinoma of the head and neck (3/9); gastrointestinal (gastric, colon, rectum, appendix, small intestine) (2/6); gallbladder carcinoma (1/3); cutaneous squamous cell carcinoma (1/3); papillary/follicular thyroid carcinoma (1/3); non‐cutaneous (mucosal, uveal, etc) (1/3); osteosarcoma (1/3); unspecified/other soft‐tissue sarcoma, liposarcoma, unspecified/other bone sarcoma, angiosarcoma, unspecified/other sarcoma, or non‐uterine leiomyosarcoma (4/12).

Of the 253 patients who had *FGFRalt*, 142 had *FGFR* fusions and 110 had *FGFR1‐3* mutations; 1 patient had both. Of the 142 patients who had *FGFR* fusions, 86 (60.6%) had *FGFR2* fusions, 48 (33.8%) had *FGFR3* fusions, and 8 (5.6%) had *FGFR1* fusions. Of the 110 patients who had *FGFR1–3* mutations, 69 (62.7%) had *FGFR2* mutations, 40 (36.4%) had *FGFR3* mutations, and 7 (6.4%) had *FGFR1* mutations. The most common *FGFR1–3* mutations were *FGFR3* S249C (27 [24.5%]), *FGFR3* R248C (9 [8.2%]), and *FGFR2* C382R (7 [6.4%]).

### 3.2 Primary Endpoint: rwOS From First‐Line Therapy Across all Tumor Types

**FIGURE 2:**
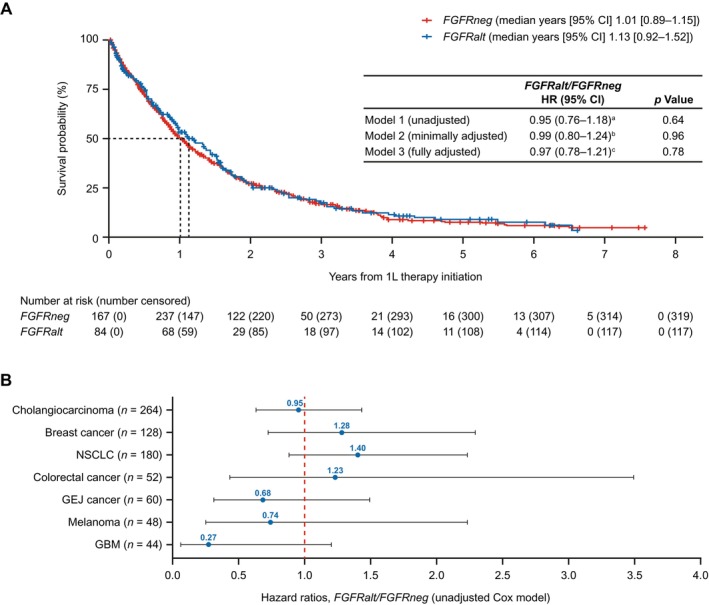
rwOS from first‐line therapy. (A) Kaplan–Meier curves across all tumor types and (B) by tumor type. A tumor‐type stratified Cox model accounting for delayed entry was used for the primary analysis. In the *FGFRneg* group, the number at risk increases from 0 to 1 year because genomic test dates for some patients occurred after first‐line initiation, and therefore those patients entered the risk set after the first‐line initiation date. 1L, first‐line; CI, confidence interval; *FGFRalt*, fibroblast growth factor receptor gene alterations; *FGFRneg*, fibroblast growth factor receptor gene without mutations or alterations; GBM, glioblastoma; GEJ, gastroesophageal junction; HR, hazard ratio; NSCLC, non‐small cell lung cancer. ^a^Unadjusted model did not include covariates. ^b^Minimally adjusted for covariates of interest: Tumor type, age at advanced diagnosis, year of advanced diagnosis (categorical), sex, and Foundation Medicine Inc. (FMI) test version (dichotomous). ^c^Fully adjusted for covariates of interest: Tumor type, age at advanced diagnosis, year of advanced diagnosis (categorical), sex, FMI test version (dichotomous), group stage, diagnosed early versus advanced (dichotomous), Charlson Comorbidity Index, and smoking status.

Median rwOS from first‐line treatment across all tumor types was 1.13 years (95% CI 0.92–1.52) in the *FGFRalt* group compared with 1.01 years (95% CI 0.89–1.15) for *FGFRneg* (Figure 2A). rwOS from first‐line therapy across all tumor types was not significantly different for patients with *FGFRalt* versus those with *FGFRneg* tumors in the primary analysis, for any of the statistical models used, including stratified Cox models fully adjusted for covariates (HR 0.97; *p* = 0.78), unadjusted stratified Cox models (HR 0.95; *p* = 0.64), and minimally adjusted, stratified Cox models (HR 0.99; *p* = 0.96). HRs < 1 correspond to a protective effect of *FGFRalt*.

### 3.3 Secondary Endpoints

#### 3.3.1 rwOS By Tumor Type and LOT

rwOS by tumor type was not significantly different between the *FGFRalt* and *FGFRneg* groups (Figure 2B). Median rwOS ranged from 0.78 years in CCA to 2.15 years in melanoma.

rwOS from second‐ and third‐line therapy was not significantly different among patients with *FGFRalt* and those with *FGFRneg* tumors (Table 3).

**TABLE 3:** Secondary endpoints (A) rwOS by LOT (B) rwTNT for *FGFRalt/FGFRneg* patients across all tumors and by subgroups of tumor types.

	Median, years (95% CI)		*FGFRalt/FGFRneg* HR (95% CI)^a^	*p*
	*FGFRalt*	*FGFRneg*		
rwOS by LOT across all tumor types				
Second‐line therapy, *n* = 508	0.75 (0.52–1.08)	0.77 (0.64–1.08)	1.06 (0.78–1.44)^b^	0.72
Third‐line therapy, *n* = 280	0.57 (0.36–1.60)	0.82 (0.58–1.11)	1.22 (0.81–1.86)^b^	0.34
rwTNT from first‐to‐second‐line therapy				
Across all tumors	0.77 (0.59–0.89)	0.73 (0.67–0.82)	1.07 (0.87–1.32)^b^	0.50
By tumor type				
Breast cancer, *n* = 128	0.45 (0.32–1.34)	0.56 (0.46–0.81)	1.17 (0.72–1.92)^c^	0.53
Cholangiocarcinoma, *n* = 264	0.59 (0.46–1.03)	0.68 (0.63–0.83)	1.13 (0.77–1.66)^c^	0.53
Colorectal cancer, *n* = 52	0.79 (0.52–NA)	0.82 (0.65–1.65)	3.88 (1.20–12.55)^c^	0.02
Gastric/esophagogastric junction cancer, *n* = 60	0.91 (0.46–NA)	0.56 (0.46–0.84)	0.27 (0.09–0.85)^c^	0.03
Melanoma, *n* = 48	NA (0.59–NA)	1.90 (0.86–NA)	1.36 (0.25–7.34)^c^	0.72
Non‐small cell lung cancer, *n* = 180	0.84 (0.68–NA)	1.26 (0.78–1.79)	1.45 (0.86–2.45)^c^	0.17

Abbreviations: CI, confidence interval; *FGFRalt*, fibroblast growth factor receptor gene alterations; *FGFRneg*, fibroblast growth factor receptor gene without mutations or alterations; HR, hazard ratio; NA, not applicable; rwTNT, real‐world time to next treatment.

^a^ Fully adjusted for covariates of interest: Tumor type, age at advanced diagnosis, year of advanced diagnosis (categorical), sex, Foundation Medicine Inc. (FMI) test version (dichotomous), group stage, diagnosed early versus advanced (dichotomous), Charlson Comorbidity Index, and smoking status.

^b^ A tumor‐type stratified Cox model accounting for delayed entry.

^c^ A standard Cox model not accounting for delayed entry.

#### 3.3.2 rwTNT Across all Tumor Types, by Tumor Type, and by LOT

rwTNT from first‐ to second‐line therapy was not significantly different among patients in the *FGFRalt* and *FGFRneg* groups across all tumor types (HR 1.07; 95% CI 0.87–1.32; *p* = 0.5) (Table 3). Median rwTNT from first‐ to second‐line therapy was 0.77 years for *FGFRalt* compared with 0.73 years for *FGFRneg*. rwTNT from first‐ to second‐line therapy was significantly different among *FGFRalt* patients compared with *FGFRneg* patients with colorectal cancer, with patients with *FGFRalt* having worse outcomes than those who were *FGFRneg* (HR 3.88; 95% CI 1.20–12.55; *p* = 0.02), whereas better outcomes were seen among patients with *FGFRalt* gastric/esophagogastric junction cancer (HR 0.27; 95% CI 0.09–0.85; *p* = 0.03) compared with those who were *FGFRneg*.

Additional secondary endpoints, sensitivity analyses, and genomic landscape co‐alterations are described in Data S1 and shown in Figures S2–S4 and Tables S7–S14.

## 4 Discussion

No statistically significant differences in rwOS were observed between matched patients with advanced *FGFRalt* and *FGFRneg* solid tumors treated with non–FGFR‐targeted therapy. The HR for rwOS between *FGFRalt* and *FGFRneg* patients for the primary endpoint was 0.97 (*p* = 0.78), and alternative statistical models yielded results consistent with those seen in the primary analysis. These results indicate that, in the real‐world setting, patients whose tumors are driven by FGFR signaling have outcomes similar to those of patients whose tumors have different genetic drivers (for tumor types included in this analysis). Kaplan–Meier survival curves for both groups were similar; median rwOS was 1.13 years from first‐line therapy for patients with *FGFRalt* and 1.01 years from first‐line therapy for patients who were *FGFRneg*. Statistically significant differences in rwOS by individual tumor type were not observed between the *FGFRalt* and *FGFRneg* groups, although the analysis was not powered for a tumor‐specific comparison. These data suggest that patients with *FGFRalt* have similarly poor OS in the advanced or metastatic setting as *FGFRneg* patients, highlighting the high unmet need and potential of FGFR‐targeting agents to improve outcomes.

Although *FGFRalt* are observed in many tumor types, previous studies were limited to select tumor types. Previous studies also had methodological challenges, including small sample size and imbalances in confounders [35, 36]. These limitations underscore the need for a rigorous pan‐tumor evaluation of how *FGFRalt* impact prognosis in patients with advanced/metastatic disease.

The present study has several key strengths: it was a large observational comparative study with the primary objective of investigating the prognostic value of pathogenic *FGFRalt* versus *FGFRneg* in patients with advanced solid tumors in a statistically robust manner. Also, the definition of *FGFRalt* was normalized to those patients in whom FGFR is most likely to be a driving event in cancer development and progression. The study was protocol driven and designed with statistical power to evaluate any differences in rwOS of *FGFRalt* patients compared with *FGFRneg* patients. The study utilized a number of statistical methods, including patient matching on known confounders, delayed entry modeling of survival outcomes, and covariate‐adjusted stratified Cox modeling, to minimize bias and ensure rigor.

A limitation of this study is the exclusion of patients with UC, thus limiting the generalizability of the results. However, UC has been studied previously, with similar results showing no significant difference observed in OS when comparing *FGFRalt* and *FGFRneg* [37]. In addition, the FoundationOne CDx test versions did not assess *FGFR4* alterations, which may have led to misclassified *FGFRalt* status. However, given the rarity of *FGFR4* alterations (< 0.04% frequency in GENIE databases, September 2022), the number of misclassified patients was likely small. *FGFR* testing is not standard of care for most tumor types; therefore, it is likely that tumor types for which *FGFR* testing is not commonly undertaken may have been underrepresented. Comorbidity information may have been incomplete as electronic health records were not oncology specific. This study was not powered to measure tumor‐specific outcomes, and therefore conclusions regarding these outcomes are limited. Patients might have been lost to follow‐up and mortality may have been underestimated. Delayed entry was accounted for in the primary models of rwOS to avoid biasing the study results; however, delayed entry was not accounted for when modeling other outcomes, and therefore the results may have been biased if timing of genetic testing relative to outcomes differed by cohort. There was a low proportion of patients with reported TMB score data, comprising ≤ 20% of patients in each cohort when confidence in the computed score did not meet the necessary threshold. High levels of absent TMB data could lead to biased HR estimates if in reality there were differential TMB levels between the *FGFRalt* and *FGFRneg* groups.

This analysis included only patients who were treated with systemic anticancer therapy after advanced/metastatic diagnosis, and thus excluded patients who were not treated with systemic anticancer therapy after advanced/metastatic disease. These untreated patients may have had poor prognosis (e.g., not candidates for any systemic therapy due to poor performance status). Thus, the analysis may have excluded a subset of patients with poor outcomes. Only patients who were statistically matched were included in the study; differences in unmatched/matched patients resulting from unmeasured confounders may have led to biased estimation if key prognostic factors were not included in subsequent covariate‐adjusted models.

This study examined the prognostic role of *FGFRalt*, independent of other genetic alterations, on survival outcomes. The cooperative impact on tumor growth and progression of *FGFRalt* and other mutations, such as those in other canonical pathways, have not been thoroughly evaluated; for example, in solid tumors with high microsatellite instability, in which immune checkpoint inhibitors have demonstrated superior clinical outcomes in select tumor types, including colorectal cancer.

In this statistically powered and matched‐cohort real‐world analysis, there was no significant difference in rwOS between patients with *FGFRalt or FGFRneg* advanced/metastatic solid tumors; median OS was approximately 1 year in both groups. These results highlight the poor prognosis and high unmet need for novel and targeted therapies in these patient populations.

## Author Contributions

**Levon Demirdjian:** conceptualization (lead), data curation (lead), formal analysis (lead), investigation (lead), methodology (lead), project administration (equal), writing – original draft (lead), writing – review and editing (lead). **Spyros Triantos:** conceptualization (equal), formal analysis (equal), investigation (equal), methodology (equal), writing – review and editing (equal). **Kristopher Standish:** data curation (equal), formal analysis (equal), investigation (equal), methodology (equal), writing – review and editing (equal). **Shibu Thomas:** conceptualization (equal), formal analysis (equal), investigation (equal), methodology (equal), writing – review and editing (equal). **Qi Xia:** conceptualization (equal), data curation (equal), formal analysis (equal), investigation (equal), writing – review and editing (equal). **Jiarui Zhang:** conceptualization (equal), formal analysis (equal), investigation (equal), methodology (equal), writing – review and editing (equal). **Joel Greshock:** conceptualization (equal), formal analysis (equal), investigation (equal), methodology (equal), writing – review and editing (equal). **Julie Paone:** conceptualization (equal), formal analysis (equal), investigation (equal), methodology (equal), writing – review and editing (equal). **Paige Sheridan:** conceptualization (equal), formal analysis (equal), investigation (equal), methodology (equal), writing – review and editing (equal). **Shubham Pant:** formal analysis (equal), writing – review and editing (equal). **Christophe Massard:** formal analysis (equal), writing – original draft (equal), writing – review and editing (equal). **David A. Reardon:** formal analysis (equal), writing – review and editing (equal). **Yohann Loriot:** formal analysis (equal), writing – review and editing (equal). **Martin Schuler:** formal analysis (equal), writing – review and editing (equal). **Hussein Sweiti:** conceptualization (equal), formal analysis (equal), investigation (equal), methodology (equal), writing – review and editing (equal).

## Ethics Statement

To ensure privacy of the patient‐level information, all analyses were conducted only using de‐identified, commercially available patient data. Confidentiality of patient records was maintained at all times. All study reports contained aggregate data only.

## Conflicts of Interest

L.D., S.T., K.S., C.H., S.T., Q.X., J.Z., J.G., and H.S. received personal fees from Janssen during the conduct of the study. J.P. and P.S. received personal fees from Aetion during the conduct of the study. S.P. has received consulting fees from Ipsen, Janssen, Novartis, and Zymeworks; and institutional research funding from 4D Pharma, Arcus Biosciences, Astellas Pharma, Boehringer Ingelheim, Bristol Myers Squibb, Elicio Therapeutics, Janssen, Lilly, Mirati Therapeutics, NGM Biopharmaceuticals, Novartis, Purple Biotech, Rgenix, and Xencor. C.M. has received consulting fees from Amgen, Astellas Pharma, AstraZeneca, Bayer, BeiGene, Blueprint Medicines, Bristol Myers Squibb, Celgene, Debiopharm Group, Faron Pharmaceuticals, Genentech/Roche, Innate Pharma, Ipsen, Janssen, Lilly, MSD, Novartis, Orion, Pfizer, PharmaMar, Sanofi, and Taiho Pharmaceutical. D.A.R. has received financial support for advisory input from Agios, AnHeart Therapeutics, Avita Biomedical Inc., Blue Rock Therapeutics, Bristol Myers Squibb, Boston Biomedical, CureVac AG, Del Mar Pharma, DNAtrix, Hoffman‐LaRoche Ltd., Imvax, Janssen, Kiyatec, Medicenna Therapeutics, Neuvogen, Novartis, Novocure, Pyramid, Sumitomo Dainippon Pharma, Vivacitas Oncology Inc., and Y‐mabs Therapeutics. Y.L. has received consulting fees from Astellas Pharma, AstraZeneca, Bristol Myers Squibb, Immunomedics, Janssen (and institutional), MSD Oncology (and institutional), Loxo/Lilly, Pfizer/EMD Serono, Roche, and Taiho Pharmaceutical; has been reimbursed for travel, accommodations, or expenses from Astellas, AstraZeneca, Janssen Oncology, MSD Oncology, and Roche; and has received institutional research funding from Astellas Pharma, AstraZeneca, Basilea, Bristol Myers Squibb, Exelixis, Gilead Sciences, Incyte, Janssen Oncology, Merck KGaA, MSD Oncology, Nektar, Pfizer, Roche, Sanofi, and Taiho Pharmaceutical. M.S. has received consulting fees from Amgen, AstraZeneca, Boehringer Ingelheim, Bristol Myers Squibb, GlaxoSmithKline, Janssen Oncology, Merck Serono, Novartis, Roche, Sanofi, and Takeda; honoraria from Amgen, Boehringer Ingelheim, Bristol Myers Squibb, Janssen‐Cilag, and Novartis; institutional research funding from AstraZeneca and Bristol Myers Squibb; and has institutional patents, royalties, or other intellectual property for a highly sensitive method for mutation detection by PCR.

## Supporting information


Data S1.

## Data Availability

Janssen Pharmaceutical Companies of Johnson & Johnson's data sharing policy is available at https://www.janssen.com/clinical‐trials/transparency. As noted on this site, requests for study data access can be submitted through the Yale Open Data Access (YODA) project site at http://yoda.yale.edu.
