# Supplementary material for: Prognostic Impact of Oncogenic Fibroblast Growth Factor Receptor Alterations in Patients With Advanced Solid Tumors in a Real‐World Setting
Source: Cancer Med. 2025 Feb 25;14(4):e70546. doi: 10.1002/cam4.70546 (PMC11862098; doi:10.1002/cam4.70546)
Supplement: Supplementary file 1 — Data S1. [file CAM4-14-e70546-s001.docx]

**SUPPLEMENTARY METHODS**

**Data source**

De-identified comprehensive genomic profiling (CGP) data and other biomarker data from Foundation Medicine, Inc. (FMI) are directly linked to de-identified longitudinal clinical, treatment and real-world outcomes data from Flatiron Health (FH), and are available in disease-specific and disease-agnostic data models composed of US-based patients diagnosed with cancer with 3-month recency. This collaboration (referred to as the “Flatiron Health-Foundation Medicine Clinico-Genomic Database” or “FMI CGDB”) integrates FH’s clinical data platform with FMI’s CGP capabilities.

Clinical data within the CGDB are sourced from FH, a health technology company. FH’s longitudinal clinical data are primarily sourced from electronic health records. FH’s clinical data capture the patient’s oncology experience and include treatment data, real-world outcomes (such as mortality, progression, and response in select diseases), disease-specific biomarkers, and prognostic factors.

The FMI component of the CGDB consists of research-grade specimen, genomic, and other biomarker data arising from clinical testing performed by FMI, and includes all underlying genomic alterations reported to clinicians from CGP of patient tumor samples from genes interrogated by the applicable FMI assay(s). The FMI dataset includes information on the date of testing, details of each molecular alteration seen (eg, gene name, DNA change, protein change), and other specimen details. Genomic alterations that have not yet been annotated (variants of unknown significance, or “VUS” alterations) are included in the FMI database.

The CGDB includes two subsets: tumor-specific databases and the pan-tumor database. If a patient does not satisfy the criteria for inclusion in a tumor-specific database, the patient then becomes eligible for inclusion into the pan-tumor database. With this hierarchy, a patient cannot exist in both the tumor-specific database and the pan-tumor database.

**Additional exclusion criteria**

Additional exclusion criteria included: 1) patients with evidence of clinical trial drug administration prior to the initiation of first-line therapy in the advanced/metastatic disease setting; 2) patients with a gap of >60 days from last clinical activity to genomic testing^[[1]](#footnote-2)^; 3) patients with a gap of ≥90 days from advanced/metastatic diagnosis to first structured activity after advanced/metastatic diagnosis^[[2]](#footnote-3)^; 4) patients with a diagnosis of primary hematological cancer at any time; 5) patients with evidence of use of any selective fibroblast growth factor receptor (FGFR) inhibitor (erdafitinib, infigratinib, pemigatinib, rogaratinib, or futibatinib [TAS-120]) on- or off-label prior to initiation of first-line therapy in the advanced/metastatic setting.

**Secondary endpoints**

Additional secondary endpoints were included in an exploratory capacity and included (complete list below): 1) real-world overall survival (rwOS) from first-line treatment by tumor type; 2) real-world time to treatment discontinuation (rwTTD) of all cancer therapies from first-line treatment defined as the time from the start of first-line treatment to discontinuation of all cancer therapies; 3) rwTTD from first-line treatment defined as the time from the start of first-line treatment to discontinuation of first-line treatment; 4) real-world time to next treatment (rwTNT) from first- to second-line treatment. rwTTD was defined as the time from the start of a specific oncologist-defined, rule-based line of therapy (LOT) to discontinuation of all cancer therapies. rwTNT was defined as the time from the start of the current LOT to the start of the next LOT. Tumor-specific LOTs were used for all tumor types classified using tumor-specific databases (as described above). Analyses requiring a LOT other than the first-line LOT were only performed for tumor types classified using tumor-specific databases, since tumor types classified using the pan-tumor database do not have a corresponding LOT definition. All LOTs in the above specified analyses were in the advanced/metastatic setting.

All planned analyses, statistical models, and end points were prespecified in the study protocol prior to any analysis of the data.

***Complete list of secondary endpoints***

- rwOS from first line by specific tumor type
- rwOS from second line for patients with second-line therapy
- rwOS from third line for patients with third-line therapy
- rwTTD of all cancer therapies (excluding urothelial cancer-specific therapies) from first line for all patients and by specific tumor type
- rwTTD of all cancer therapies (excluding urothelial cancer-specific therapies) from second line for patients with second-line therapy
- rwTTD of all cancer therapies (excluding urothelial cancer-specific therapies) from third line for patients with second-line therapy
- rwTTD of first line for all patients and by specific tumor type
- rwTTD of second line for patients with second-line therapy
- rwTTD of third line for patients with third-line therapy
- rwTNT from first line to second line for all patients and by specific tumor type
- rwTNT from second line to third line for patients with second-line therapy
- rwTNT from third line to fourth line for patients with third-line therapy
- rwOS from second line for patients with second-line therapy by treatment type
- rwOS from third line for patient with third-line therapy by treatment type

**Mortality capture**

A composite mortality variable was developed that represents a patient’s vital status and date of death, which combines publicly available Social Security Death Index data and obituary data (processed from obituaries, funeral homes, and other sources) with the electronic health record (EHR)-derived mortality data (ie, structured date of death and abstracted information from available EHR documentation). Validation analyses performed by benchmarking mortality data against National Death Index data achieved sensitivity of 86% to 99% and specificity of 94% to 100% depending on cancer type and data model (Curtis, 2018; Zhang, 2021).

**Left truncation and immortal time bias**

The dataset was subject to left truncation and immortal time bias. Left truncation occurs when a dataset does not include patients with events that occur before a prespecified time (Klein, 2003). For example, patients who died before having genomic testing and/or did not have a second visit prior to or after 2011 would not be included in the dataset.

Patients entered the risk sets at the date of the genomic test result used to define exposure status or index date of the respective analysis, whichever occurred later. For *FGFRneg* patients, the date of the first *FGFRneg* result was used as the date of genomic testing result. For *FGFRalt* patients, the date of the first *FGFRalt* result was used as the date of genomic testing result. For patients with multiple *FGFR* test results, the date of the first result determined *FGFRalt*/*FGFRneg* cohort assignment to yield an unbiased choice of time zero (Hernan, 2016). Patients with both an *FGFRalt* result and *FGFRneg* result (17 patients in the dataset) were included in the *FGFRalt* group. A sensitivity analysis was performed excluding patients with discordant *FGFR* testing results (described below).

**Sensitivity analyses**

A series of sensitivity analyses were conducted, including: 1) a landmark analysis in the primary objective; patients were included in this analysis if they were not censored and did not have the outcome within 30 days from the start of first-line therapy and if they both had the second visit and genetic testing no more than 30 days after start of first-line therapy; 2) indexing on the start of first-line therapy for all patients, regardless of date of genomic testing results; 3) unstratified Cox models in analyses where stratified Cox model was used; 4) removal of patients with discordant test results (defined as having at least one positive and one negative *FGFR* test result; 17 patients [1.7%] in the matched cohort had discordant test results); and 5) removal of all patients with NSCLC with pathogenic somatic mutations in *EGFR* or *BRAF* V600E, or any gene fusions in *ALK*, *ROS1*, or *NTRK* (in line with the RAGNAR study). These co-alterations could have a prognostic impact on outcomes of patients harboring these alterations.

**Statistical analysis**

**Variables used for matching *FGFRalt*/*FGFRneg* patients**

The following variables were used to match *FGFRalt/FGFRneg* patients based on clinical guidance and potential to have a prognostic effect on study outcomes:

- Tumor type (tumor type is a direct determinant of overall prognosis and treatment patterns, and the prevalence and types of observed *FGFR* alterations differ by type)
- Age at advanced/metastatic disease
- Date of advanced/metastatic disease (this may be associated with available genomic testing and treatment options, which may have impacted study outcomes)
- Sex
- FMI test version. FMI test version is the specific version of the FMI genomic test platform. Different versions of the assay were available at different times. Imbalances in the FMI test version may have resulted in biased effect estimates. These qualifying alterations were tested against the FMI test version as part of the FMI CDx assay validation. Certain FMI test versions did not assess *FGFR4* alterations. Therefore, patients in the comparator cohort may have been incorrectly classified as *FGFRneg* if they had an undetected *FGFR4* alteration; however, given the rarity of *FGFR4* alterations (*FGFR4* Y367C mutations had <0.04% frequency, GENIE databases accessed September 2022), the number of misclassified patients was expected to be small.

**Censoring rules**

- Patients were followed from the index date until the first of 1) outcome (death), 2) FGFR inhibitor use, 3) end of data, or 4) last clinical activity in the absence of evidence of death (to determine last known alive date). For all non–death-related outcomes, death was included as a censoring event.

**SUPPLEMENTARY RESULTS**

**Additional secondary endpoints**

**rwOS from first-line therapy by type of FGFR alteration**

The hazard ratios (HRs) per *FGFR* status were also not significantly different for models specifically testing *FGFR* mutations or *FGFR* fusions versus their wild-type comparators (Table S6).

**Across all tumor types**

rwTTD of all cancer therapies from first-line therapy was not significantly different for *FGFRalt* patients compared with *FGFRneg* patients across all tumor types (HR 1.11; 95% confidence interval [CI] 0.79–1.58; *p* = 0.54) (Table S7). rwTTD of first-line therapy was not significantly different for *FGFRalt* patients compared with *FGFRneg* patients across all tumor types (HR 1.13; 95% CI 0.92–1.38; *p* = 0.24). Median rwTTD of first-line therapy was 0.41 years (95% CI 0.34–0.49) for *FGFRalt* patients compared with 0.46 years (95% CI 0.42–0.52) for *FGFRneg* patients.

**Subgroups of tumor types**

rwTTD of all cancer therapies from first-line therapy was not significantly different for *FGFRalt* patients compared with *FGFRneg* patients by subgroups of tumor types (Table S7). rwTTD of first-line therapy was significantly different for *FGFRalt* patients compared with *FGFRneg* patients with cholangiocarcinoma (HR 1.43; 95% CI 1.01–2.02; *p* = 0.04), with *FGFRalt* patients having worse outcomes than *FGFRneg* patients.

**Subgroups of line settings**

rwTTD of all cancer therapies from second- and third-line therapy was not significantly different for *FGFRalt* patients compared with *FGFRneg* patients (Table S8). rwTTD of second- and third-line therapy was not significantly different for *FGFRalt* patients compared with *FGFRneg* patients. rwTNT from third-line therapy to fourth-line therapy was significantly different for *FGFRalt* patients compared with *FGFRneg* patients (among patients who received at least three lines of therapy), with a median rwTNT of 0.37 years for *FGFRalt* patients compared with 0.68 years for *FGFRneg* patients (HR 2.09; 95% CI 1.37–3.18; *p* < 0.001).

**Sensitivity analyses: Primary endpoint**

rwOS from first-line therapy across all tumor types was not significantly different for *FGFRalt* patients compared with *FGFRneg* patients in a sensitivity analysis that did not use delayed-entry models (Cox model stratified by tumor type; rwOS HR 0.83; *p* = 0.09) (Figure S2). This trend for a potential *FGFRalt*-protective effect was partially driven by an increased time between the first-line therapy initiation date and genomic testing among *FGFRalt* patients compared with *FGFRneg* patients (Poisson *p* < 0.001). *FGFRalt* patients had a median time from first-line therapy to *FGFR* testing of 98 days, compared with *FGFRneg* patients, with a median time of 73 days from first-line therapy to *FGFR* testing. Not accounting for this delayed entry induces survivorship bias and creates the appearance of better survival among *FGFRalt* patients as their survival times are increased by a median of 25 days, a period over which they could not have experienced a death event (immortal time) (Yadav, 2021). The delayed entry model accounts for the artificial increase in survival.

rwOS from first-line therapy across all tumor types was also not significantly different comparing *FGFRalt* versus *FGFRneg* patients for other sensitivity analyses (Table S9).

**Sensitivity analyses: Secondary endpoints across all tumor types**

Sensitivity analyses using non-delayed entry for secondary endpoints of rwTTD of all cancer therapies from first-line therapy, rwTTD of first-line therapy, and rwTNT from first- to second-line therapy were not significantly different between *FGFRalt* and *FGFRneg* patients (Table S10).

**Analysis of rwOS in glioblastoma patients**

Based on our observation of an apparent though non-significant protective effect of *FGFRalt* in patients with glioblastoma (ie, rwOS HR of 0.27 favoring the *FGFRalt* vs. *FGFRneg* group), we conducted a post hoc analysis to determine the extent to which co-occurring alterations might be contributing to observed survival differences. In an analysis of single nucleotide variants (SNVs) after filtering for genes with a frequency of >10% and for non-synonymous somatic mutations, only alterations in *EGFR* had differential frequencies between *FGFRalt* and *FGFRneg* patients, occurring more frequently in *FGFRneg* glioblastoma patients compared with patients with *FGFRalt* (Figure S3). Of 44 patients with glioblastoma, 0 of 11 *FGFRalt* patients had *EGFR* alterations compared with 9 of 33 *FGFRneg* patients. This was considered notable as prior studies showed a significant reduction in OS in patients with glioblastoma having *EGFR^A289D/T/V^* mutations (Binder, 2018). However, subsequent removal of all *FGFRneg* patients with *EGFR* SNVs and their *FGFRalt* matches did not meaningfully alter rwOS results, resulting in a similarly protective trend for *FGFRalt* (Table S11).

A second exploratory analysis to measure the effect of *EGFR* SNVs on rwOS, irrespective of *FGFR* status, yielded effect estimates for *EGFR* that were not statistically significant (Table S12). Finally, a pathogenicity score was assigned to each patient with glioblastoma using the number of pathogenic or likely pathogenic alterations (excluding *FGFR* alterations) present in the tumor sample (American College of Medical Genetics [ACMG] guidelines were used to annotate mutation pathogenicity). The pathogenicity score was not significantly associated with rwOS, nor did inclusion of the score meaningfully alter the observed HR for FGFR status on rwOS (Table S13).

More detailed review of the Kaplan-Meier curves for the GBM cohort showed that the apparent protective effect of *FGFRalt* appeared attributable to two long-lived *FGFRalt* patients, neither of whose tumors harbored any pathogenic or likely pathogenic alterations (Figure S4). Excluding these two patients from Cox models removed the observed trend in the original glioblastoma-specific rwOS analysis (rwOS HR 1.35; *p* = 0.68).

**Table S1** Sample size and power calculations.

| **Power** | **HR^a^** | **Ratio of *FGFRalt* to comparator** | **Total deaths** | **Participants, *n*** | ***FGFRalt* participants, *n*** |
| --- | --- | --- | --- | --- | --- |
| 90% | 0.75 | 1:3 | 681 | 959 | 240 |
| 90% |  | 1:4 | 794 | 1110 | 222 |
| 80% | 0.75 | 1:3 | 509 | 716 | 179 |
| 80% |  | 1:4 | 593 | 830 | 166 |
| 90% | 1.30 | 1:3 | 819 | 1242 | 311 |
| 90% |  | 1:4 | 954 | 1457 | 292 |
| 80% | 1.30 | 1:3 | 612 | 928 | 232 |
| 80% |  | 1:4 | 713 | 1089 | 218 |

Abbreviations: *FGFRalt*, fibroblast growth factor receptor gene alterations; *FGFRneg*, fibroblast growth factor receptor gene without alterations or mutations; HR, hazard ratio; rwOS, real-world overall survival.

^a^ HRs >1 correspond to worse rwOS outcomes among patients with *FGFRalt* relative to the *FGFRneg* cohort.

Matching ratios larger than 1:4 offer minimal gains in efficiency (Austin, 2010). The power calculations were computed using the Schoenfeld formula (Schoenfeld, 1983).

**Table S2** Demographic and baseline characteristics of *FGFRalt* and broad cohort of *FGFRneg*

*(matched + unmatched)* patients across all tumors.

| **Characteristic** | ***FGFRalt* (*n* = 253)** | ***FGFRneg***  **(matched +**  **unmatched)**  ***N* = 20,512** |
| --- | --- | --- |
| **Age at advanced/metastatic diagnosis** | | |
| **Median (range), years** | 62 (18–84) | 64 (55–72) |
| **Year of advanced/metastatic diagnosis** | | |
| 2011  2012  2013  2014  2015  2016  2017  2018  2019  2020 | 0 (0.0)  6 (2.4)  11 (4.3)  14 (5.5)  25 (9.9)  30 (11.9)  36 (14.2)  50 (19.8)  51 (20.2)  30 (11.9) | 291 (1.4)  510 (2.5)  986 (4.8)  1620 (7.9)  2215 (11)  2548 (12)  3145 (15)  3480 (17)  3327 (16)  2390 (12) |
| **ECOG PS at advanced/metastatic diagnosis** | | |
| 0  1  ≥2  Unknown | 81 (32.0)  80 (31.6)  25 (9.9)  67 (26.5) | 2941 (14)  2594 (13)  561 (2.7)  14,416 (70) |
| **Practice type** | | |
| Academic  Community | 28 (11.1)  225 (88.9) | 2234 (11)  18,278 (89) |
| **Sex** | | |
| Male  Female | 113 (44.7) 140 (55.3) | 11,047 (54)  9465 (46) |
| **Race** | | |
| White  Black or African American  Asian  Hispanic or Latino  Other  Unknown | 183 (72.3) 21 (8.3) 4 (1.6)  1 (0.4)  30 (11.9)  14 (5.5) | 14304 (70)  1417 (6.9)  444 (2.2)  43 (0.2)  3017 (15)  1287 (6.3) |

Abbreviations: ECOG PS, Eastern Cooperative Oncology Group performance status; *FGFRalt*, fibroblast growth factor receptor gene alterations; *FGFRneg*, fibroblast growth factor receptor gene without mutations or alterations.

All values are n (%) except where otherwise noted.

Variables used in the matching algorithm were balanced between *FGFRalt*/*FGFRneg* groups in the analyses of all patients but may have become unbalanced in analyses of subgroups of patients who initiated second- and third-line treatment, and patient subgroups based on treatment type for specific tumor types. Imbalances were accounted for by including the variables used for matching in multivariate covariate adjustment.

**Table S3** Additional demographic and baseline characteristics of *FGFRalt/FGFRneg* patients across all tumors.

| **Characteristic** | ***FGFRalt* (*n* = 253)** | ***FGFRneg***  **(*n* = 759)** | **Absolute standardized difference in means/proportions** |
| --- | --- | --- | --- |
| **Demographic characteristics among all tumors** | | | |
| Year of advanced/metastatic diagnosis  2011  2012  2013  2014  2015  2016  2017  2018  2019  2020 | 0 (0.0)  6 (2.4)  11 (4.3)  14 (5.5)  25 (9.9)  30 (11.9)  36 (14.2)  50 (19.8)  51 (20.2)  30 (11.9) | 3 (0.4)  9 (1.2)  33 (4.3)  49 (6.5)  69 (9.1)  90 (11.9)  122 (16.1)  161 (21.2)  121 (15.9)  102 (13.4) | 0.09  0.09  0.00  0.04  0.03  0.00  0.05  0.04  0.11  0.05 |
| Practice type  Academic  Community | 28 (11.1)  225 (88.9) | 72 (9.5)  687 (90.5) | 0.05 |
| Payer category  Commercial health plan  Medicare  Medicaid  Self-pay  Other  Unknown  Missing | 43 (17.0)  7 (2.8)  7 (2.8)  3 (1.2)  17 (6.7)  21 (8.3)  168 (66.4) | 120 (15.8)  34 (4.5)  4 (0.5)  9 (1.2)  41 (5.4)  67 (8.8)  502 (66.1) | 0.03  0.09  0.18  0.00  0.06  0.02  0.01 |
| **Clinical characteristics (among all tumors)** | | | |
| Therapies by medication type (prior to initiation of first-line therapy in the advanced/metastatic setting)  Immuno + chemo + hormonal  Chemo + hormonal  Chemo + immuno  Hormonal + immuno  Chemo only  Hormonal only  Immuno only  None | 0 (0.0)  3 (1.2)  0 (0.0)  0 (0.0)  9 (3.6)  8 (3.2)  1 (0.4)  232 (91.7) | 1 (0.1)  22 (2.9)  4 (0.5)  0 (0.0)  18 (2.4)  19 (2.5)  1 (0.1)  694 (91.4) | 0.05  0.12  0.10  0.00  0.07  0.04  0.05  0.01 |
| Evidence of any PD-L1 testing  Yes  No | 17 (6.7)  236 (93.3) | 48 (6.3)  711 (93.7) | 0.02 |
| Time from initial diagnosis to advanced/metastatic disease diagnosis  Mean (SD), days | 362.63 (740.99) | 396.63 (891.23) | 0.04 |
| **Clinical characteristics at baseline** | | | |
| Hemoglobin, g/dL  <10 g/dL  ≥10 g/dL  Missing | 19 (7.5)  195 (77.1)  39 (15.4) | 67 (8.8)  536 (70.6)  156 (20.6) | 0.05  0.15  0.13 |
| Creatinine clearance  Median (IQR) (mg/dL) | 205 (81.0)  0.81 (0.69–1.04) | 605 (79.7)  0.83 (0.70–1.00) | 0.03 |
| Serum albumin  Median (IQR) (g/L) | 195 (77.1)  40.0 (36.0–43.0) | 572 (75.4)  40.0 (37.0–42.0) | 0.07 |
| CCI components  Myocardial infarction  Congestive heart failure  Peripheral vascular disease  Cerebrovascular disease  Dementia  Chronic pulmonary disease  Connective tissue disease  Moderate to severe renal disease  Diabetes mellitus without end organ damage  Diabetes mellitus with end organ damage  Hemiplegia/paraplegia  Peptic ulcer disease  Mild chronic liver disease  Moderate or severe liver disease  AIDS/HIV | 23 (9.1)  20 (7.9)  18 (7.1)  17 (6.7)  2 (0.8)  63 (24.9)  6 (2.4)  19 (7.5)  49 (19.4)  9 (3.6)  3 (1.2)  16 (6.3)  26 (10.3)  3 (1.2)  2 (0.8) | 43 (5.7)  29 (3.8)  38 (5.0)  45 (5.9)  3 (0.4)  184 (24.2)  31 (4.1)  60 (7.9)  147 (19.4)  33 (4.3)  10 (1.3)  42 (5.5)  54 (7.1)  12 (1.6)  2 (0.3) | 0.13  0.17  0.09  0.03  0.05  0.02  0.10  0.01  0  0.04  0.01  0.03  0.11  0.03  0.07 |

Abbreviations: AIDS, acquired immunodeficiency syndrome; CCI, Charlson Comorbidity Index; *FGFRalt*, fibroblast growth factor receptor gene alterations; *FGFRneg*, fibroblast growth factor receptor gene without alterations or mutations; HIV, human immunodeficiency virus; IQR, interquartile range; PD-L1, programmed death ligand 1; SD, standard deviation.

All values are n (%) except where otherwise noted.

**Table S4** Demographic and baseline characteristics of *FGFRalt/FGFRneg* patients across all tumors – by mutation or fusion status.

|  | **Mutations** | | | **Fusions** | | |
| --- | --- | --- | --- | --- | --- | --- |
| **Characteristic** | ***FGFRalt* (*n* = 110)** | ***FGFRneg***  **(*n* = 330)** | **Absolute standardized difference in means/ proportions** | ***FGFRalt* (*n* = 142)** | ***FGFRneg***  **(*n* = 426)** | **Absolute standardized difference in means/ proportions** |
| **Demographic characteristics among all tumors** | | | | | | |
| Age at advanced/metastatic diagnosis  Median (range), years  <65 years  ≥65 years | 65 (58-73)  54 (49.0)  56 (51.0) | 65 (57-72)  163 (49.4)  167 (50.6) | 0.03  0.00 | 59 (53-67)  93 (65.5)  49 (34.5) | 60 (54-68)  263 (61.7)  163 (38.3) | 0.09  0.00 |
| Sex  Male  Female | 59 (53.6) 51 (46.4) | 177 (53.6) 153 (46.4) | 0.00 | 54 (38.0) 88 (62.0) | 162 (38.0) 264 (62.0) | 0.00 |
| Race  White  Black or African American  Asian  Hispanic or Latino  Other  Unknown | 79 (71.8)  8 (7.3)  2 (1.8)  0 (0.0)  14 (12.7)  7 (6.4) | 219 (66.4)  28 (8.5)  11 (3.3)  2 (0.6)  49 (14.8)  21 (6.4) | 0.12  0.05  0.10  0.11  0.06  0.00 | 103 (72.5) 13 (9.2)  2 (1.4)  1 (0.7)  16 (11.3)  7 (4.9) | 283 (66.4)  25 (5.9)  7 (1.6)  0 (0.0)  76 (17.8)  35 (8.2) | 0.13  0.13  0.02  0.12  0.19  0.13 |
| Smoking history  Yes  No  Unknown | 48 (43.6) 62 (56.4) 0 (0.0) | 122 (37.0)  208 (63.0)  0 (0.0) | 0.14  0.14  NA | 64 (45.1) 78 (54.9) 0 (0.0) | 197 (46.2)  227 (53.3)  2 (0.5) | 0.02  0.03  0.10 |
| **Clinical characteristics among all tumors** | | | | | | |
| Group stage at initial diagnosis  Group stage 0  Group stage 1  Group stage 2  Group stage 3  Group stage 4  Missing | 9 (8.2) 14 (12.7) 26 (23.6)  51 (46.4)  10 (9.1)  0 (0.0) | 34 (10.3)  33 (10.0)  57 (17.3)  162 (49.2)  43 (13.1)  1 (0.3) | 0.07  0.09  0.16  0.06  0.13  0.08 | 9 (6.3) 12 (8.5) 25 (17.6)  77 (54.2)  19 (13.4)  0 (0.0) | 17 (4.0)  54 (12.7)  50 (11.8)  223 (52.6)  80 (18.9)  2 (0.5) | 0.11  0.14  0.17  0.04  0.15  0.10 |
| Tumor mutational burden (version 1)^a^  Median (IQR) | 27 (24.5)  10.0 (3.2–43.1) | 77 (23.3)  6.3 (3.5–16.3) | 0.56 | 15 (10.6)  3.8 (1.3–5.7) | 72 (16.9)  3.5 (1.7–6.1) | 0.18 |
| Tumor mutational burden (version 2)^b^  Median (IQR) | 24 (21.8)  10.7 (5.7–69.3) | 70 (21.2)  7.0 (3.5–18.8) | 0.63 | 16 (11.3)  2.6 (1.3–6.0) | 60 (14.1)  3.8 (2.5–6.5) | 0.29 |
| Time from advanced/ metastatic disease to first systemic treatment  Median (IQR) | 108 (98.2)  39.5 (17.0–64.5) | 314 (95.2)  32.0 (18.0–57.0) | 0.12 | 135 (95.1)  29.0 (18.0–43.5) | 398 (93.4)  29.0 (16.0–48.0) | 0.02 |
| **Clinical characteristics at baseline** | | | | | | |
| ECOG PS  0  1  2  3  4 | 36 (43.4) 35 (42.2) 9 (10.8)  3 (3.6)  0 (0.0) | 114 (47.5)  100 (41.7)  20 (8.3)  6 (2.5)  0 (0.0) | 0.04  0.03  0.08  0.06  NA | 45 (44.1) 44 (43.1) 10 (9.8)  3 (2.9)  0 (0.0) | 121 (44.5)  112 (41.2)  30 (11.0)  8 (2.9)  1 (0.4) | 0.07  0.10  0.00  0.02  0.07 |
| Modified CCI score  Median (IQR) | 1.0 (0.0–2.0) | 1.0 (0.0–2.0) | 0.18 | 1.0 (0.0–2.0) | 1.0 (0.0–2.0) | 0.00 |

Abbreviations: CCI, Charlson Comorbidity Index; ECOG PS, Eastern Cooperative Oncology Group performance status; *FGFRalt*, fibroblast growth factor receptor gene alterations; *FGFRneg*, fibroblast growth factor receptor gene without mutations or alterations; IQR, interquartile range.

^a^ Version 1 approximates the tumor mutational burden score from running all samples and is biomarker-harmonized to incorporate recent scientific understanding.

^b^ Version 2 approximates clinically reported values and the variant annotations used are based on latest scientific understanding at the time of reporting.

All values are n (%) except where otherwise noted.

**Table S5** Most common treatment regimens across tumor types.

|  | ***FGFRalt*** | ***FGFRneg*** | ***FGFRalt*** | ***FGFRneg*** | ***FGFRalt*** | ***FGFRneg*** |
| --- | --- | --- | --- | --- | --- | --- |
|  | **First-line therapy** | | **Second-line therapy** | | **Third-line therapy** | |
| **Cholangiocarcinoma** | ***n* = 66** | ***n* = 198** | ***n* = 42** | ***n* = 122** | ***n* = 22** | ***n* = 59** |
| Cisplatin, gemcitabine  Fluorouracil, leucovorin, oxaliplatin  Clinical study drug  Capecitabine  Fluorouracil, irinotecan, leucovorin | 39 (59.1)  8 (12.1)  0 (0.0)  1 (1.5)  0 | 100 (50.5)  21 (10.6)  0 (0.0)  8 (4.0)  3 (1.5) | 7 (16.7)  8 (19.0)  7 (16.7)  1 (2.4)  1 (2.4) | 11 (9.0)  27 (22.1)  15 (12.3)  13 (10.7)  8 (6.6) | 1 (4.5)  2 (9.1)  4 (18.2)  3 (13.6)  4 (18.2) | 1 (1.7)  8 (13.6)  3 (5.1)  5 (8.5)  8 (13.6) |
| **Breast cancer** | ***n* = 32** | ***n* = 96** | ***n* = 24** | ***n* = 71** | ***n* = 20** | ***n* = 49** |
| Carboplatin, gemcitabine  Letrozole, palbociclib  Fulvestrant, palbocicib  Clinical study drug  Capecitabine  Exenestane | 4 (12.5)  4 (12.5)  4 (12.5)  0 (0.0)  0 (0.0)  1 (3.1) | 0 (0.0)  12 (12.5)  10 (10.4)  0 (0.0)  4 (4.2)  0 (0.0) | 0 (0.0)  0 (0.0)  1 (4.2)  3 (12.5)  2 (8.3)  0 (0.0) | 2 (2.8)  5 (7.0)  5 (7.0)  3 (4.2)  5 (7.0)  0 (0.0) | 0 (0.0)  0 (0.0)  1 (5.0)  0 (0.0)  5 (25.0)  3 (15.0) | 1 (2.0)  1 (2.0)  3 (6.1)  2 (4.1)  6 (12.2)  1 (2.0) |

| **Non–small-cell lung cancer** | ***n* = 45** | ***n* = 135** | ***n* = 22** | ***n* = 66** | ***n* = 12** | ***n* = 28** |
| --- | --- | --- | --- | --- | --- | --- |
| Carboplatin, paclitaxel  Carboplatin, gemcitabine  Pembrolizumab  Carboplatin, pembrolizumab, pemetrexed  Nivolumab  Clinical study drug  Docetaxel, ramucirumab  Carboplatin, pemetrexed | 9 (20.0)  5 (11.1)  5 (11.1)  4 (8.9)  3 (6.7)  0 (0.0)  1 (2.2)  2 (4.4) | 18 (13.3)  3 (2.2)  26 (19.3)  19 (14.1)  5 (3.7)  0 (0.0)  0 (0.0)  9 (6.7) | 2 (9.1)  0 (0.0)  3 (13.6)  1 (4.5)  6 (27.3)  3 (13.6)  0 (0.0)  0 (0.0) | 1 (1.5)  1 (1.5)  4 (6.1)  4 (6.1)  16 (24.2)  3 (4.5)  1 (1.5)  4 (6.1) | 0 (0.0)  1 (8.3)  0 (0.0)  0 (0.0)  2 (16.7)  2 (16.7)  2 (16.7)  1 (8.3) | 0 (0.0)  3 (10.7)  1 (3.6)  0 (0.0)  2 (7.1)  2 (7.1)  0 (0.0)  3 (10.7) |
| **Colorectal cancer** | ***n* = 13** | ***n* = 39** | - | - | - | - |
| FOLFOX, bevacizumab  FOLFOX  FOLFIRI, bevacizumab | 5 (38.5)  1 (7.7)  1 (7.7) | 11 (28.2)  7 (17.9)  6 (15.4) | -  -  - | -  -  - | -  -  - | -  -  - |

| **Gastric/esophagogastric  junction cancer** | ***n* = 15** | ***n* = 45** | ***n* = 10** | ***n* = 35** | - | - |
| --- | --- | --- | --- | --- | --- | --- |
| FOLFOX  Paclitaxel, ramucirumab  FOLFOX, trastuzumab  CAPEOX  Carboplatin, paclitaxel  Pembrolizumab  FOLFIRI | 8 (53.3)  3 (20.0)  2 (13.3)  0 (0.0)  1 (6.7)  1 (6.7)  0 (0.0) | 14 (31.1)  1 (2.2)  5 (11.1)  6 (13.3)  5 (11.1)  0 (0.0)  0 (0.0) | 0 (0.0)  2 (20.0)  1 (10.0)  1 (10.0)  0 (0.0)  5 (50.0)  1 (10.0) | 3 (8.6)  10 (28.6)  0 (0.0)  0 (0.0)  1 (2.9)  4 (11.4)  4 (11.4) | -  -  -  -  -  -  - | -  -  -  -  -  - |
| **Melanoma** | ***n* = 12** | ***n* = 36** | - | - | - | - |
| Ipilimumab, nivolumab  Nivolumab  Pembrolizumab | 4 (33.3)  4 (33.3)  2 (16.7) | 15 (41.7)  10 (27.8)  8 (22.2) | -  -  - | -  -  - | -  -  - | -  -  - |

Abbreviations: *FGFRalt*, fibroblast growth factor receptor gene alterations; *FGFRneg*, fibroblast growth factor receptor gene without alterations or mutations.

All values are n (%) except where otherwise noted. Data reported are for ≥ 10% frequency in any group for the stated regimen
(ie, treatments could have been used in other combinations).

**Table S6** Frequency of most common mutations and fusions in *FGFRalt* patients among specific tumors.

| ***FGFRalt* by tumor type^a^** | ***n* (%)** |
| --- | --- |
| Cholangiocarcinoma  *FGFR2*-F276C  *FGFR2-BICC1*  Breast cancer  *FGFR2-*K659N  *FGFR3-TACC3*  Non-small cell lung cancer  *FGFR3-*S249C  *FGFR3-TACC3*  Colorectal cancer  *FGFR2-*L770V  *FGFR3-TACC3*  Gastric/esophagogastric junction cancer  *FGFR2-*C382R  *FGFR3-TACC3*  Melanoma  *FGFR2-*E731K  NA  Glioblastoma  *FGFR1*-K656E  *FGFR2*-S252L  *FGFR3-TACC3* | 6 (9.1) 19 (28.8)  3 (9.4)  5 (15.6)  13 (28.9)  14 (31.1)  2 (15.4)  2 (15.4)  2 (13.3)  6 (40)  5 (41.7)  NA^b^  1 (9.1)  1 (9.1)  9 (81.8) |

Abbreviations: *FGFRalt*, fibroblast growth factor receptor gene alterations; NA, not applicable.

^a^ Under each tumor type is listed the most common *FGFR* mutation followed by the most common *FGFR* fusion.

^b^ There were no patients with melanoma who had an *FGFR* fusion.

**Table S7** Real-world survival from first-line therapy across all tumor types – medians and HRs for all models (unadjusted, minimally adjusted, and fully adjusted for covariates) and by *FGFRalt* types (overall, *FGFRalt* mutation only, *FGFRalt* fusion only). A tumor-type stratified Cox model accounting for delayed entry was used for the primary analysis.

|  | **Median, years (95% CI)** | | ***FGFRalt/FGFRneg***  **HR (95% CI)** | ***p* Value** |
| --- | --- | --- | --- | --- |
|  | ***FGFRalt* mutations and fusions** | |  |  |
|  | ***FGFRalt*** | ***FGFRneg*** |  |  |
| Model 1 (unadjusted) | 1.13 (0.92–1.52) | 1.01 (0.89–1.15) | 0.95 (0.76–1.18)^a^ | 0.64 |
| Model 2 (minimally adjusted) |  |  | 0.99 (0.80–1.24)^b^ | 0.96 |
| Model 3 (fully adjusted) |  |  | 0.97 (0.78–1.21)^c^ | 0.78 |
|  | ***FGFRalt* mutations vs. *FGFRneg* matches** | |  |  |
|  | ***FGFRalt*** | ***FGFRneg*** |  |  |
| Model 1 (unadjusted) | 0.98 (0.63–1.55) | 1.20 (0.97–1.61) | 1.18 (0.84–1.66)^a^ | 0.33 |
| Model 2 (minimally adjusted) |  |  | 1.39 (0.97–1.97)^b^ | 0.07 |
| Model 3 (fully adjusted) |  |  | 1.31 (0.91–1.89)^c^ | 0.14 |
|  | ***FGFRalt* fusions vs. *FGFRneg* matches** | |  |  |
|  | ***FGFRalt*** | ***FGFRneg*** |  |  |
| Model 1 (unadjusted) | 1.34 (0.97–1.65) | 0.89 (0.78–1.08) | 0.84 (0.63–1.13)^a^ | 0.26 |
| Model 2 (minimally adjusted) |  |  | 0.89 (0.66–1.20)^b^ | 0.45 |
| Model 3 (fully adjusted) |  |  | 0.85 (0.63–1.16)^c^ | 0.31 |

Abbreviations: CI, confidence interval; *FGFRalt*, fibroblast growth factor receptor gene alterations; *FGFRneg*, fibroblast growth factor receptor gene without alterations or mutations; HR, hazard ratio.

^a^ Unadjusted model did not include covariates.

^b^ Minimally adjusted for covariates of interest: tumor type, age at advanced diagnosis, year of advanced diagnosis (categorical), sex, and Foundation Medicine Inc. (FMI) test version (dichotomous).

^c^ Fully adjusted for covariates of interest: tumor type, age at advanced diagnosis, year of advanced diagnosis (categorical), sex, FMI test version (dichotomous), group stage, diagnosed early versus advanced (dichotomous), Charlson Comorbidity Index, and smoking status.

**Table S8** Secondary endpoints for *FGFRalt*/*FGFRneg* patients across all tumors and by subgroups of tumor types – all models (unadjusted, minimally adjusted, and fully adjusted for covariates).

|  | | ***FGFRalt/FGFRneg***  **HR (95% CI)** | | ***p* Value** |
| --- | --- | --- | --- | --- |
| **Across all tumors** | | | | |
| Real-world time to discontinuation off all cancer therapies from first-line therapy | Model 1 (unadjusted) | 1.23 (0.88–1.72)^a,b^ | 0.23 | |
|  | Model 2 (minimally adjusted) | 1.16 (0.82–1.63)^b,c^ | 0.40 | |
|  | Model 3 (fully adjusted) | 1.11 (0.79–1.58)^b,d^ | 0.54 | |
| Real-world time to treatment discontinuation of first-line therapy | Model 1 (unadjusted) | 1.13 (0.93–1.37)^a,b^ | 0.22 | |
|  | Model 2 (minimally adjusted) | 1.15 (0.94–1.40)^b,c^ | 0.18 | |
|  | Model 3 (fully adjusted) | 1.13 (0.92–1.38)^b,c^ | 0.24 | |
| Real-world time to next treatment from first- to second-line therapy | Model 1 (unadjusted) | 1.06 90.86–1.29)^a,e^ | 0.60 | |
|  | Model 2 (minimally adjusted) | 1.07 (0.87–1.31)^c,e^ | 0.54 | |
|  | Model 3 (fully adjusted) | 1.07 (0.87–1.32)^d,e^ | 0.50 | |
| **Subgroups of tumor types** | | | | |
| **rwOS** | | | | |
| Breast cancer, *n* = 128 | Model 1 (unadjusted) | 1.28 (0.72–2.29)^a,f^ | 0.40 | |
|  | Model 2 (minimally adjusted) | 1.28 (0.71–2.32)^c,f^ | 0.41 | |
|  | Model 3 (fully adjusted) | 1.28 (0.68–2.42)^d,f^ | 0.44 | |
| Cholangiocarcinoma, *n* = 264 | Model 1 (unadjusted) | 0.95 (0.63–1.43)^a,f^ | 0.81 | |
|  | Model 2 (minimally adjusted) | 1.01 (0.67–1.54)^c,f^ | 0.95 | |
|  | Model 3 (fully adjusted) | 0.98 (0.64–1.50)^d,f^ | 0.92 | |
| Colorectal cancer, *n* = 52 | Model 1 (unadjusted) | 1.23 (0.43–3.49)^a,f^ | 0.70 | |
|  | Model 2 (minimally adjusted) | 2.65 (0.59–11.90)^c,f^ | 0.20 | |
|  | Model 3 (fully adjusted) | 3.44 (0.61–19.34)^d,f^ | 0.16 | |
| Gastric/esophagogastric junction cancer, *n* = 60 | Model 1 (unadjusted) | 0.68 (0.31–1.49)^a,f^ | 0.33 | |
|  | Model 2 (minimally adjusted) | 0.67 (0.29–1.51)^c,f^ | 0.33 | |
|  | Model 3 (fully adjusted) | 0.54 (0.19–1.51)^d,f^ | 0.24 | |
| Glioblastoma multiforme, *n* = 44 | Model 1 (unadjusted) | 0.27 (0.06–1.20)^a,f^ | 0.09 | |
|  | Model 2 (minimally adjusted) | 0.25 (0.05–1.22)^c,f^ | 0.09 | |
|  | Model 3 (fully adjusted) | 0.60 (0.11–3.23)^c,f^ | 0.56 | |
| Melanoma, *n* = 48 | Model 1 (unadjusted) | 0.74 (0.25–2.23)^a,f^ | 0.60 | |
|  | Model 2 (minimally adjusted) | 0.76 (0.24–2.36)^c,f^ | 0.63 | |
|  | Model 3 (fully adjusted) | 0.52 (0.10–2.66)^d,f^ | 0.44 | |
| Non-small cell lung cancer, *n* = 80 | Model 1 (unadjusted) | 1.40 (0.88–2.23)^a,f^ | 0.16 | |
|  | Model 2 (minimally adjusted) | 1.43 (0.88–2.32)^c,f^ | 0.15 | |
|  | Model 3 (fully adjusted) | 1.49 (0.91–2.45)^d,f^ | 0.11 | |
| **rwTTD of all cancer therapies from first-line therapy** | | | | |
| Breast cancer, *n* = 128 | Model 1 (unadjusted) | 1.59 (0.54–4.66)^a,g^ | 0.40 | |
|  | Model 2 (minimally adjusted) | 1.00 (0.27–3.70)^c,g^ | 0.99 | |
|  | Model 3 (fully adjusted) | 1.39 (0.31–6.28)^d,g^ | 0.67 | |
| Cholangiocarcinoma, *n* = 264 | Model 1 (unadjusted) | 1.60 (0.93–2.73)^a,g^ | 0.09 | |
|  | Model 2 (minimally adjusted) | 1.47 (0.85–2.55)^c,g^ | 0.17 | |
|  | Model 3 (fully adjusted) | 1.52 (0.86–2.68)^d,g^ | 0.15 | |
| Colorectal cancer, *n* = 52 | Model 1 (unadjusted) | 0.52 (0.11–2.54)^a,g^ | 0.42 | |
|  | Model 2 (minimally adjusted) | 0.45 (0.08–2.55)^c,g^ | 0.36 | |
|  | Model 3 (fully adjusted) | 0.003 (0.00001–0.91)^d,g^ | 0.05 | |
| Gastric/esophagogastric junction cancer, *n* = 60 | Model 1 (unadjusted) | 0.68 (0.19–2.45)^a,g^ | 0.56 | |
|  | Model 2 (minimally adjusted) | 0.32 (0.06–1.71)^c,g^ | 0.18 | |
|  | Model 3 (fully adjusted) | 0.49 (0.05–4.40)^d,g^ | 0.52 | |
| Melanoma, *n* = 48 | Model 1 (unadjusted) | 1.62 (0.40–6.50)^a,g^ | 0.50 | |
|  | Model 2 (minimally adjusted) | 1.38 (0.32–5.90)^c,g^ | 0.66 | |
|  | Model 3 (fully adjusted) | 1.36 (0.15–12.24)^d,g^ | 0.78 | |
| Non-small cell lung cancer, *n* = 180 | Model 1 (unadjusted) | 0.75 (0.31–1.81)^a,g^ | 0.52 | |
|  | Model 2 (minimally adjusted) | 0.88 (0.36–2.19)^c,g^ | 0.79 | |
|  | Model 3 (fully adjusted) | 0.67 (0.26–1.77)^d,g^ | 0.42 | |
| **rwTTD of first-line therapy** | | | | |
| Breast cancer, *n* = 128 | Model 1 (unadjusted) | 1.15 (0.72–1.83)^a,g^ | 0.56 | |
|  | Model 2 (minimally adjusted) | 1.11 (0.69–1.81)^c,g^ | 0.66 | |
|  | Model 3 (fully adjusted) | 1.19 (0.73–1.96)^d,g^ | 0.49 | |
| Cholangiocarcinoma, *n* = 264 | Model 1 (unadjusted) | 1.43 (1.01–2.02)^a,g^ | 0.04 | |
|  | Model 2 (minimally adjusted) | 1.41 (0.99–2.00)^c,g^ | 0.06 | |
|  | Model 3 (fully adjusted) | 1.43 (0.99–2.07)^d,g^ | 0.06 | |
| Colorectal cancer, *n* = 52 | Model 1 (unadjusted) | 0.80 (0.38–1.70)^a,g^ | 0.57 | |
|  | Model 2 (minimally adjusted) | 1.51 (0.57–4.05)^c,g^ | 0.41 | |
|  | Model 3 (fully adjusted) | 1.61 (0.56–4.63)^d,g^ | 0.38 | |
| Gastric/esophagogastric junction cancer, *n* = 60 | Model 1 (unadjusted) | 0.83 (0.41–1.64)^a,g^ | 0.58 | |
|  | Model 2 (minimally adjusted) | 0.83 (0.39–1.77)^c,g^ | 0.64 | |
|  | Model 3 (fully adjusted) | 0.42 (0.16–1.10)^d,g^ | 0.08 | |
| Melanoma, *n* = 48 | Model 1 (unadjusted) | 0.86 (0.32–2.34)^a,g^ | 0.77 | |
|  | Model 2 (minimally adjusted) | 0.81 (0.29–2.30)^c,g^ | 0.70 | |
|  | Model 3 (fully adjusted) | 1.23 (0.32–4.68)^d,g^ | 0.76 | |
| Non-small cell lung cancer, *n* = 180 | Model 1 (unadjusted) | 1.38 (0.87–2.19)^a,g^ | 0.17 | |
|  | Model 2 (minimally adjusted) | 1.43 (0.88–2.32)^c,g^ | 0.15 | |
|  | Model 3 (fully adjusted) | 1.31 (0.80–2.13)^d,g^ | 0.28 | |
| **rwTNT from first- to second-line therapy** | | | | |
| Breast cancer, *n* = 128 | Model 1 (unadjusted) | 1.13 (0.71–1.79)^a,g^ | 0.62 | |
|  | Model 2 (minimally adjusted) | 1.11 (0.69–1.80)^c,g^ | 0.67 | |
|  | Model 3 (fully adjusted) | 1.17 (0.72–1.92)^d,g^ | 0.53 | |
| Cholangiocarcinoma, *n* = 264 | Model 1 (unadjusted) | 1.18 (0.82–1.69)^a,g^ | 0.37 | |
|  | Model 2 (minimally adjusted) | 1.17 (0.81–1.70)^c,g^ | 0.40 | |
|  | Model 3 (fully adjusted) | 1.13 (0.77–1.66)^d,g^ | 0.53 | |
| Colorectal cancer, *n* = 52 | Model 1 (unadjusted) | 0.93 (0.42–2.07)^a,g^ | 0.86 | |
|  | Model 2 (minimally adjusted) | 1.89 (0.69–5.17)^c,g^ | 0.22 | |
|  | Model 3 (fully adjusted) | 3.88 (1.20–12.55)^d,g^ | 0.02 | |
| Gastric/esophagogastric junction cancer, *n* = 60 | Model 1 (unadjusted) | 0.64 (0.31–1.31)^a,g^ | 0.22 | |
|  | Model 2 (minimally adjusted) | 0.65 (0.30–1.43)^c,g^ | 0.29 | |
|  | Model 3 (fully adjusted) | 0.27 (0.09–0.85)^d,g^ | 0.03 | |
| Melanoma, *n* = 48 | Model 1 (unadjusted) | 0.74 (0.21–2.62)^a,g^ | 0.64 | |
|  | Model 2 (minimally adjusted) | 0.89 (0.23–3.43)^c,g^ | 0.86 | |
|  | Model 3 (fully adjusted) | 1.36 (0.25–7.34)^d,g^ | 0.72 | |
| Non-small cell lung cancer, *n* = 180 | Model 1 (unadjusted) | 1.45 (0.89–2.38)^a,g^ | 0.14 | |
|  | Model 2 (minimally adjusted) | 1.62 (0.97–2.70)^c,g^ | 0.07 | |
|  | Model 3 (fully adjusted) | 1.45 (0.86–2.45)^d,g^ | 0.17 | |

Abbreviations: CI, confidence interval; *FGFRalt*, fibroblast growth factor receptor gene alterations; *FGFRneg*, fibroblast growth factor receptor gene without alterations or mutations; HR, hazard ratio; rwOS, real-world overall survival; rwTNT, real-world time to next treatment; rwTTD, real-world time to treatment discontinuation.

^a^ Unadjusted for covariates.

^b^ A tumor-type stratified Cox model not accounting for delayed entry.

^c^ Minimally adjusted for covariates of interest: Tumor type, age at advanced diagnosis, year of advanced diagnosis (categorical), sex, and Foundation Medicine Inc. (FMI) test version (dichotomous).

^d^ Fully adjusted for covariates of interest: Tumor type, age at advanced diagnosis, year of advanced diagnosis (categorical), sex, FMI test version (dichotomous), group stage, diagnosed early versus advanced (dichotomous), Charlson Comorbidity Index, and smoking status.

^e^ A tumor-type stratified Cox model accounting for delayed entry.

^f^ A standard Cox model accounting for delayed entry that does not include strata by tumor type.

^g^ A standard Cox model not accounting for delayed entry.

**Table S9** Subgroups of line setting.

|  | **Median, years (95% CI)** | | ***FGFRalt/FGFRneg***  **HR (95% CI)^a^** | ***p* Value** |
| --- | --- | --- | --- | --- |
|  | ***FGFRalt*** | ***FGFRneg*** |  |  |
| rwTTD of all cancer therapies from second- and third-line therapy across all tumor types  Second-line therapy, *n* = 530  Third-line therapy, *n* = 280 | 5.56 (1.83–NA)  4.60 (1.68–NA) | NA (NA–NA) NA (NA–NA) | 1.02 (0.63–1.65)^b^  1.19 (0.58–2.46)^b^ | 0.94  0.63 |
| rwTTD of second- and third-line therapy across all tumor types  Second-line therapy, *n* = 530  Third-line therapy, *n* = 290 | 0.50 (0.45–0.69)  0.29 (0.25–0.63) | 0.40 (0.35–0.50) 0.45 (0.36–0.52) | 0.78 (0.58–1.05)^b^  1.29 (0.86–1.93)^b^ | 0.11  0.22 |
| rwTNT from second- to third-line therapy and third-line to fourth-line therapy across all tumor types  Second-line therapy, *n* = 530  Third-line therapy, *n* = 290 | 0.71 (0.56–0.86)  0.37 (0.29–0.70) | 0.65 (0.52–0.82) 0.68 (0.52–0.97) | 1.00 (0.74–1.35)^b^  2.09 (1.37–3.18)^b^ | 0.98  < 0.001 |

Abbreviations: CI, confidence interval; *FGFRalt*, fibroblast growth factor receptor gene alterations; *FGFRneg*, fibroblast growth factor receptor gene without alterations or mutations; HR, hazard ratio; NA, not applicable; rwTNT, real-world time to next treatment; rwTTD, real-world time to treatment discontinuation.

^a^ Fully adjusted for covariates of interest: Tumor type, age at advanced diagnosis, year of advanced diagnosis (categorical), sex, Foundation Medicine Inc. test version (dichotomous), group stage, diagnosed early versus advanced (dichotomous), Charlson Comorbidity Index, and smoking status.

^b^ A tumor-type stratified Cox model accounting for delayed entry.

**Table S10** Sensitivity analyses of rwOS from first-line therapy across all tumor types.

|  | **Median, years (95% CI)** | | **Model (adjustment for covariates)** | ***FGFRalt*/*FGFRneg***  **HR (95% CI)** | ***p* Value** |
| --- | --- | --- | --- | --- | --- |
|  | ***FGFRalt*** | ***FGFRneg*** |  |  |  |
| Delayed entry, standard^a^ | - | - | Model 1 (unadjusted) | 0.98 (0.79–1.20)^b^ | 0.82 |
|  |  |  | Model 2 (minimally adjusted) | 1.02 (0.82–1.26)^c^ | 0.87 |
|  |  |  | Model 3 (fully adjusted) | 0.99 (0.80–1.22)^d^ | 0.91 |
| Non-delayed entry, stratified^e^ | 1.88 (1.58-2.54) | 1.63 (1.48-1.80) | Model 1 (unadjusted) | 0.83 (0.67–1.02)^b^ | 0.08 |
|  |  |  | Model 2 (minimally adjusted) | 0.84 (0.68–1.04)^c^ | 0.11 |
|  |  |  | Model 3 (fully adjusted) | 0.83 (0.67–1.03)^d^ | 0.09 |
| Landmark analysis, stratified^f^ | 1.34 (0.98-1.91) | 1.26 (1.06-1.62) | Model 1 (unadjusted) | 1.05 (0.77–1.44)^b^ | 0.75 |
|  |  |  | Model 2 (minimally adjusted) | 1.17 (0.85–1.62)^c^ | 0.33 |
|  |  |  | Model 3 (fully adjusted) | 1.14 (0.82–1.59)^d^ | 0.43 |
| Delayed entry, stratified (removing patients with discordant test results)^g^ | 1.13 (0.92-1.52) | 1.01 (0.89-1.15) | Model 1 (unadjusted) | 0.94 (0.75–1.17)^b^ | 0.57 |
|  |  |  | Model 2 (minimally adjusted) | 0.98 (0.79–1.23)^c^ | 0.87 |
|  |  |  | Model 3 (fully adjusted) | 0.96 (0.77–1.20)^d^ | 0.70 |
| Non-small cell lung cancer^h^ | 0.89 (0.46-1.53) | 0.98 (0.67-1.61) | Model 1 (unadjusted) | 1.50 (0.93–2.44)^b^ | 0.10 |
|  |  |  | Model 2 (minimally adjusted) | 1.52 (0.92–2.51)^c^ | 0.10 |
|  |  |  | Model 3 (fully adjusted) | 1.65 (0.98–2.80)^d^ | 0.06 |
| Activity event within 90 days^i^ | 1.13 (0.92-1.53) | 0.98 (0.87-1.13) | Model 1 (unadjusted) | 0.98 (0.77–1.23)^b^ | 0.083 |
|  |  |  | Model 2 (minimally adjusted) | 1.01 (0.79–1.28)^c^ | 0.96 |
|  |  |  | Model 3 (fully adjusted) | 0.99 (0.78–1.26)^d^ | 0.97 |

Abbreviations: CI, confidence interval; *FGFRalt*, fibroblast growth factor receptor gene alterations; *FGFRneg*, fibroblast growth factor receptor gene without alterations or mutations; HR, hazard ratio.

^a^ Standard models correspond to Cox models that do not include strata by tumor type.

^b^ Unadjusted for covariates.

^c^ Minimally adjusted for covariates of interest: Tumor type, age at advanced diagnosis, year of advanced diagnosis (categorical), sex, and Foundation Medicine Inc. (FMI) test version (dichotomous).

^d^ Fully adjusted for covariates of interest: Tumor type, age at advanced diagnosis, year of advanced diagnosis (categorical), sex, FMI test version (dichotomous), group stage, diagnosed early versus advanced (dichotomous), Charlson Comorbidity Index, and smoking status.

^e^ Stratified refers to stratified by tumor type.

^f^ In the landmark analysis, patients were included if they were not censored and did not have the outcome within 30 days from the start of first-line therapy and if they both had the second visit and genetic testing no more than 30 days after the start of first-line therapy.

^g^ Discordant test results refer to patients who had both an *FGFRalt* test result and a *FGFRneg* test result.

^h^ Did not include patients with pathogenic somatic mutations in *EGFR* or *BRAF* V600E, or any gene fusions in *ALK*, *ROS1*, or *NTRK*.

^i^ Did not include patients who did not have ≥1 activity event within 90 days prior to their index date.

**Table S11** Sensitivity secondary end points for *FGFRalt*/*FGFRneg* patients across all tumors (non-delayed entry – standard sensitivity analysis).

|  | **Model (adjustment for covariates)** | ***FGFRalt/FGFRneg*HR (95% CI)** | ***p* Value** |
| --- | --- | --- | --- |
| rwTTD of all cancer therapies from first-line therapy | Model 1 (unadjusted) | 1.24 (0.89–1.73)^a^ | 0.21 |
|  | Model 2 (minimally adjusted) | 1.17 (0.83–1.64)^b^ | 0.38 |
|  | Model 3 (fully adjusted) | 1.12 (0.80–1.59)^c^ | 0.51 |
| rwTTD of first-line therapy | Model 1 (unadjusted) | 1.09 (0.90–1.31)^a^ | 0.40 |
|  | Model 2 (minimally adjusted) | 1.09 (0.90–1.32)^b^ | 0.38 |
|  | Model 3 (fully adjusted) | 1.08 (0.89–1.31)^c^ | 0.45 |
| rwTNT from first- to second-line therapy | Model 1 (unadjusted) | 1.05 (0.86–1.29)^a^ | 0.61 |
|  | Model 2 (minimally adjusted) | 1.06 (0.86–1.30)^b^ | 0.58 |
|  | Model 3 (fully adjusted) | 1.07 (0.87–1.31)^c^ | 0.52 |

Abbreviations: CI, confidence interval; *FGFRalt*, fibroblast growth factor receptor gene alterations; *FGFRneg*, fibroblast growth factor receptor gene without alterations or mutations; HR, hazard ratio; rwTNT, real-world time to next treatment; rwTTD, real-world time to treatment discontinuation.

^a^ Unadjusted for covariates.

^b^ Minimally adjusted for covariates of interest: Tumor type, age at advanced diagnosis, year of advanced diagnosis (categorical), sex, and Foundation Medicine Inc. (FMI) test version (dichotomous).

^c^ Fully adjusted for covariates of interest: Tumor type, age at advanced diagnosis, year of advanced diagnosis (categorical), sex, FMI test version (dichotomous), group stage, diagnosed early versus advanced (dichotomous), Charlson Comorbidity Index, and smoking status.

**Table S12** rwOS in patients with glioblastoma minus all *FGFRalt* and *FGFRneg* patients who had *EGFR* alterations.

| **Model (adjustment for covariates)** | **Patients with glioblastoma (*n* = 26)^a^** | |
| --- | --- | --- |
|  | ***FGFRalt/FGFRneg***  **HR (95% CI)** | ***p* Value** |
| Model 1 (unadjusted) | 0.30 (0.06–1.44)^b^ | 0.13 |
| Model 2 (minimally adjusted) | 0.07 (0.01–0.74)^c^ | 0.03 |
| Model 3 (fully adjusted) | 0.07 (0.00–1.59)^d^ | 0.10 |

Abbreviations: CI, confidence interval; *FGFRalt*, fibroblast growth factor receptor gene alterations; *FGFRneg*, fibroblast growth factor receptor gene without alterations or mutations; HR, hazard ratio; rwOS, overall survival.

^a^ Excluding 18 patients who had any *EGFR* alterations (*n* = 9 *FGFRneg* patients), or who were matched to patients with *EGFR* alterations (*n* = 9 *FGFRalt* patients).

^b^ Unadjusted for covariates.

^c^ Minimally adjusted for covariates of interest: Tumor type, age at advanced diagnosis, year of advanced diagnosis (categorical), sex, and Foundation Medicine Inc. (FMI) test version (dichotomous).

^d^ Fully adjusted for covariates of interest: Tumor type, age at advanced diagnosis, year of advanced diagnosis (categorical), sex, FMI test version (dichotomous), group stage, diagnosed early versus advanced (dichotomous), Charlson Comorbidity Index, and smoking status.

**Table S13** Effect of *EGFR* on rwOS in patients with glioblastoma regardless of *FGFR* status.

| **Model (adjustment for covariates)** | **Patients with glioblastoma (*n* = 29)^a^** | |
| --- | --- | --- |
|  | ***EGFRalt/EGFRneg***  **HR (95% CI)** | ***p* Value** |
| Model 1 (unadjusted) | 2.85 (0.56–14.45)^b^ | 0.21 |
| Model 2 (minimally adjusted) | 7.39 (0.61–88.94)^c^ | 0.12 |
| Model 3 (fully adjusted) | 2.35 (0.17–32.59)^d^ | 0.52 |

Abbreviations: CI, confidence interval; *EGFR*, epidermal growth factor receptor; *EGFRalt*, epidermal growth factor receptor gene alterations; *EGFRneg*, epidermal growth factor receptor gene without alterations or mutations; *FGFR*, fibroblast growth factor receptor; HR, hazard ratio; rwOS, real-world overall survival.

^a^ Excluding patients (*n* = 15) with any *EGFR* copy number variants.

^b^ Unadjusted for covariates.

^c^ Minimally adjusted for covariates of interest: Tumor type, age at advanced diagnosis, year of advanced diagnosis (categorical), sex, and Foundation Medicine Inc. (FMI) test version (dichotomous).

^d^ Fully adjusted for covariates of interest: Tumor type, age at advanced diagnosis, year of advanced diagnosis (categorical), sex, FMI test version (dichotomous), group stage, diagnosed early versus advanced (dichotomous), Charlson Comorbidity Index, and smoking status.

**Table S14** Effect of pathogenic alteration number on rwOS in patients with glioblastoma**.**

|  | **Patients with glioblastoma (*n* = 41^a^)** | | | |
| --- | --- | --- | --- | --- |
|  | **HR (95% CI)** | | | ***p* Value** |
| Model 1 (unadjusted) | - | | | - |
| *FGFRalt*/*FGFRneg* | 0.27 (0.06–1.28)^b^ | | | 0.10 |
| Sum of pathogenic alterations | 1.01 (0.80–1.28) | | | 0.94 |
|  |  | | |  |
| **Number of pathogenic alterations** | **Number (%) of patients** | ***FGFRneg*** | ***FGFRalt*** | |
| 0 | 15 (37) | 10 (31) | 5 (56) | |
| 1 | 10 (24) | 8 (25) | 2 (22) | |
| 2 | 5 (12) | 5 (16) | 0 (0) | |
| 3 | 3 (7) | 3 (9) | 0 (0) | |
| 4 | 5 (12) | 3 (9) | 2 (22) | |
| 5 | 2 (5) | 2 (6) | 0 (0) | |
| 6 | 1 (2) | 1 (3) | 0 (0) | |

Abbreviations: CI, confidence interval; *FGFRalt*, fibroblast growth factor receptor gene alterations; *FGFRneg*, fibroblast growth factor receptor gene without alterations or mutations; HR, hazard ratio; rwOS, real-world overall survival.

^a^ Three glioblastoma patients had a delay date that occurred after their censoring date and were excluded from delayed entry analyses.

^b^ Unadjusted for covariates.

**Figure S1** *FGFR* mutation and fusion specifications as defined in the RAGNAR study (Pant, 2023). *FGFR*, fibroblast growth factor receptor.

***FGFR* mutation specifications:**

- Defined as protein-coding single nucleotide variant and insertions or deletions (indels) (listed in the box below). Copy number gains or gene-level amplifications are not eligible. *FGFR* mutations annotated as germline in local reports or patients presenting with a hereditary condition/disorder associated with a germline *FGFR* mutation are not eligible for enrollment in the absence of a qualifying *FGFR* mutation or fusion.

***FGFR* fusion specifications:**

- The presence of an intact *FGFR* kinase domain.
- *FGFR* fusion with a 3-prime partner; the *FGFR* portion of the fusion must involve exon 17 or greater (≥17).
- *FGFR* fusion with a 5-prime partner; the *FGFR* portion of the fusion must involve exon 11 or less (≤11).
- Have a named *FGFR* fusion partner gene (self-fusions or rearrangements, eg, *FGFR*-*FGFR*, are not eligible).

| **Gene** | **Variant** |  | **Gene** | **Variant** |  | **Gene** | **Variant** |  | **Gene** | **Variant** |
| --- | --- | --- | --- | --- | --- | --- | --- | --- | --- | --- |
| *FGFR1* | K656E |  | *FGFR2* | C390YS |  | *FGFR3* | M528I |  | *FGFR4* | Y367C |
| *FGFR1* | R189C |  | *FGFR2* | E565G |  | *FGFR3* | K650T |  |  |  |
| *FGFR1* | S125L |  | *FGFR2* | E565Q |  | *FGFR3* | S371G |  |  |  |
| *FGFR1* | P150S |  | *FGFR2* | S252L |  | *FGFR3* | K650N |  |  |  |
|  |  |  | *FGFR2* | C382F |  | *FGFR3* | G380E |  |  |  |
|  |  |  | *FGFR2* | P253L |  | *FGFR3* | E627D |  |  |  |
|  |  |  | *FGFR2* | R251Q |  | *FGFR3* | Y373N |  |  |  |
|  |  |  | *FGFR2* | A389T |  | *FGFR3* | Y373H |  |  |  |
|  |  |  | *FGFR2* | S252P |  | *FGFR3* | D641N |  |  |  |
|  |  |  | *FGFR2* | R210Q |  | *FGFR3* | S249Y |  |  |  |
|  |  |  | *FGFR2* | S252T |  | *FGFR3* | A391V |  |  |  |
|  |  |  | *FGFR2* | R203H |  | *FGFR3* | S249F |  |  |  |
|  |  |  | *FGFR2* | S252A |  | *FGFR3* | S371R |  |  |  |
|  |  |  | *FGFR2* | S351C |  | *FGFR3* | R248H |  |  |  |
|  |  |  | *FGFR2* | Y340C |  | *FGFR3* | G370S |  |  |  |
|  |  |  | *FGFR2* | G338R |  | *FGFR3* | R669Q |  |  |  |
|  |  |  | *FGFR2* | S354C |  | *FGFR3* | P250R |  |  |  |
|  |  |  | *FGFR2* | L617F |  | *FGFR3* | Y278C |  |  |  |
|  |  |  | *FGFR2* | W290R |  | *FGFR3* | L324V |  |  |  |
|  |  |  | *FGFR2* | L550F |  | *FGFR3* | S84L |  |  |  |
|  |  |  | *FGFR2* | M535I |  | *FGFR3* | R750C |  |  |  |
|  |  |  | *FGFR2* | Y308C |  | *FGFR3* | S433C |  |  |  |
|  |  |  | *FGFR2* | E777* |  | *FGFR3* | K650Q |  |  |  |
|  |  |  | *FGFR2* | K641R |  | *FGFR3* | S371C |  |  |  |
|  |  |  | *FGFR2* | T370R |  | *FGFR3* | S249C |  |  |  |
|  |  |  | *FGFR2* | W72C |  | *FGFR3* | G370C |  |  |  |
|  |  |  | *FGFR2* | K526E |  | *FGFR3* | R248C |  |  |  |
|  |  |  | *FGFR2* | D304N |  | *FGFR3* | Y373C |  |  |  |
|  |  |  | *FGFR2* | K659M |  |  |  |  |  |  |
|  |  |  | *FGFR2* | S267P |  |  |  |  |  |  |
|  |  |  | *FGFR2* | E731K |  |  |  |  |  |  |
|  |  |  | *FGFR2* | M537I |  |  |  |  |  |  |
|  |  |  | *FGFR2* | F276C |  |  |  |  |  |  |
|  |  |  | *FGFR2* | I547V |  |  |  |  |  |  |
|  |  |  | *FGFR2* | E565A |  |  |  |  |  |  |
|  |  |  | *FGFR2* | V395D |  |  |  |  |  |  |
|  |  |  | *FGFR2* | W290C |  |  |  |  |  |  |
|  |  |  | *FGFR2* | R678G |  |  |  |  |  |  |
|  |  |  | *FGFR2* | E777K |  |  |  |  |  |  |
|  |  |  | *FGFR2* | C382R |  |  |  |  |  |  |
|  |  |  | *FGFR2* | S372C |  |  |  |  |  |  |
|  |  |  | *FGFR2* | A315T |  |  |  |  |  |  |
|  |  |  | *FGFR2* | D101Y |  |  |  |  |  |  |
|  |  |  | *FGFR2* | Y375C |  |  |  |  |  |  |
|  |  |  | *FGFR2* | E219K |  |  |  |  |  |  |
|  |  |  | *FGFR2* | L770* |  |  |  |  |  |  |
|  |  |  | *FGFR2* | L770V |  |  |  |  |  |  |
|  |  |  | *FGFR2* | K659N |  |  |  |  |  |  |

**Figure S2** Kaplan-Meier curves for rwOS from first-line therapy across all tumor types (sensitivity analysis – non-delayed entry). CI, confidence interval; *FGFRalt*, fibroblast growth factor receptor gene alterations; *FGFRneg*, fibroblast growth factor receptor gene without alterations or mutations; HR, hazard ratio.


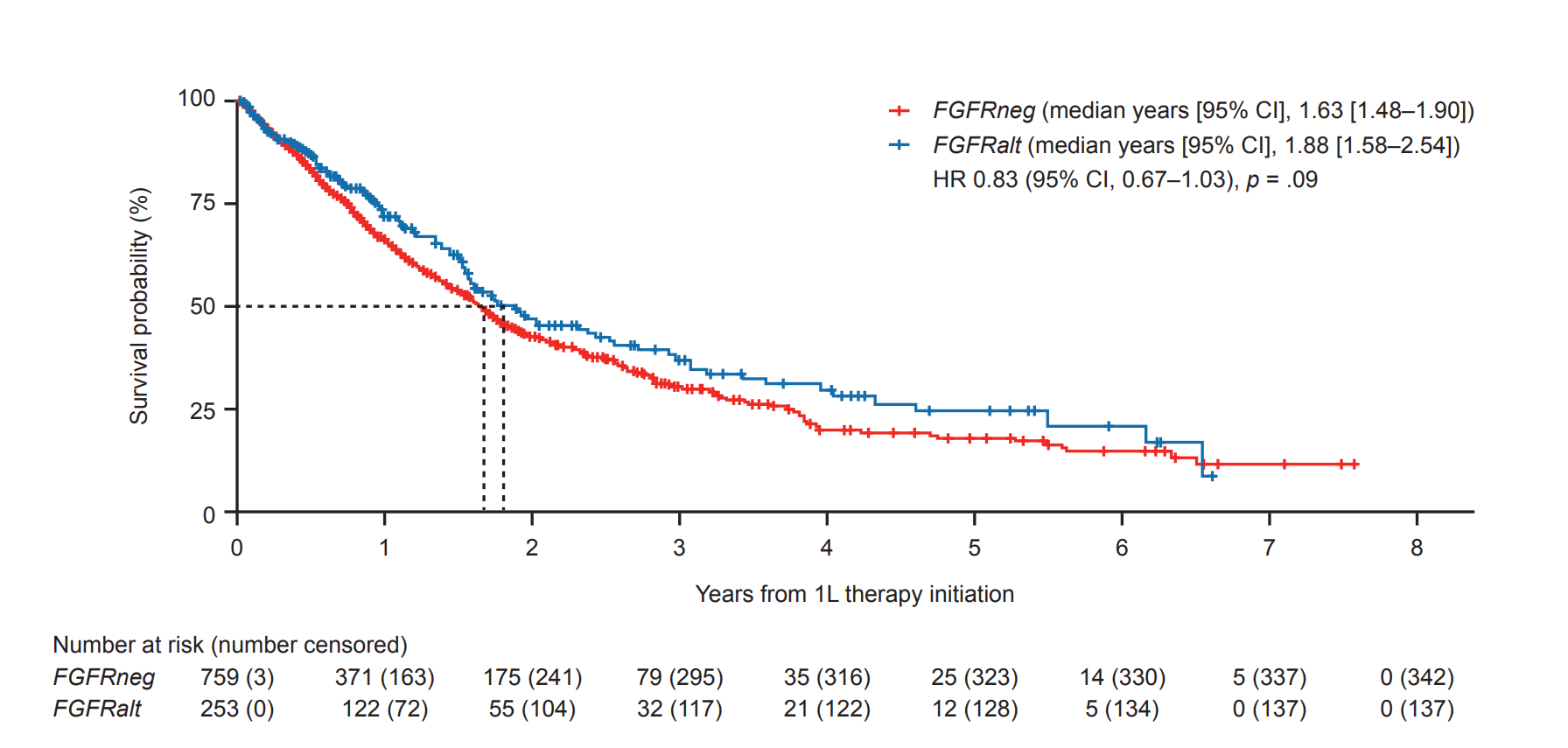


**Figure S3** Analysis of genomic SNVs in patients with glioblastoma (*FGFRalt* vs. *FGFRneg*). CI, confidence interval; *FGFRalt*, fibroblast growth factor receptor gene alterations; *FGFRneg*, fibroblast growth factor receptor gene without alterations or mutations; SNV, single nucleotide variant.


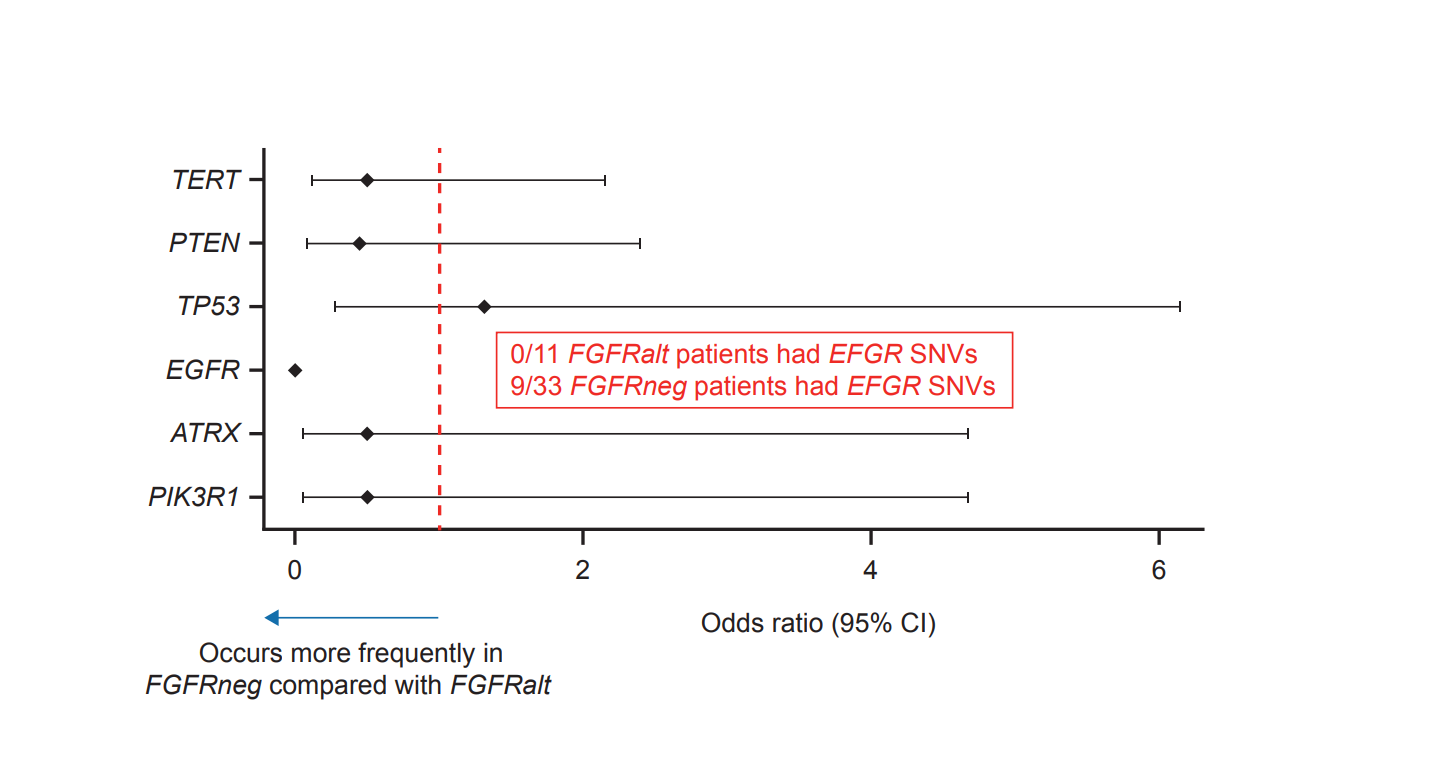


**Figure S4** Kaplan-Meier curves of patients with glioblastoma by *FGFR* status – two long-lived patients with *FGFRalt* (illustrated as triangles in figure) following first-line therapy may explain the apparent protective *FGFRalt* effect on rwOS. *FGFR*, fibroblast growth factor receptor; *FGFRalt*, fibroblast growth factor receptor gene alterations; *FGFRneg*, fibroblast growth factor receptor gene without alterations or mutations; rwOS, real-world overall survival.


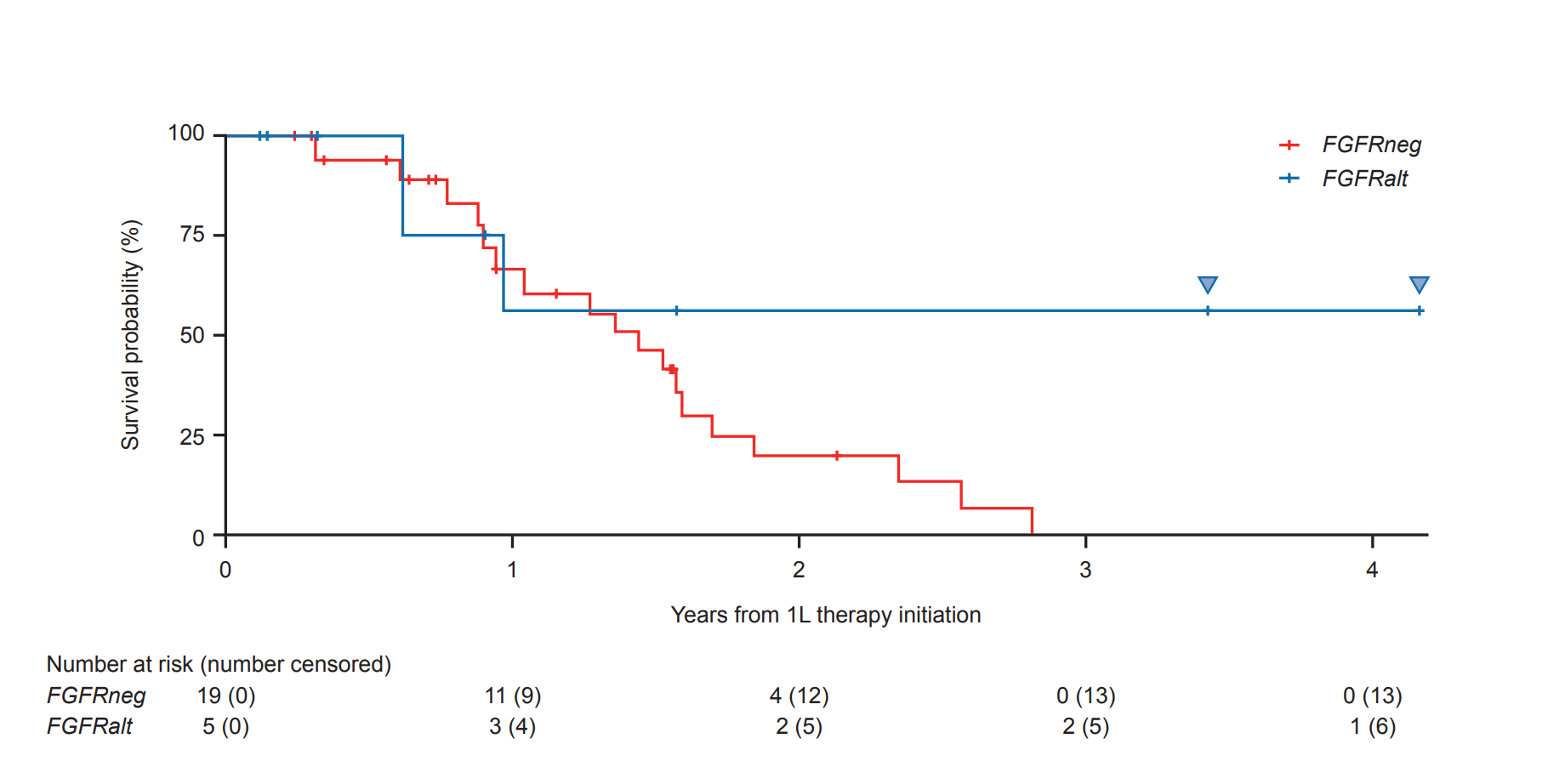


**Supplementary References**

Austin PC. Statistical criteria for selecting the optimal number of untreated subjects matched to each treated subject when using many-to-one matching on propensity score. *Am J Epidemiol*. 2010;172(9):1092-1097. doi: 10.1093/aje/kwq224.

Binder ZA, Thorne AH, Bakas S, et al. Epidermal Growth Factor Receptor Extracellular Domain Mutations in Glioblastoma Present Opportunities for Clinical Imaging and Therapeutic Development. *Cancer Cell*. 2018;34(1):163-177 e167. doi: 10.1016/j.ccell.2018.06.006.

Curtis MD, Griffith SD, Tucker, M, et al. Development and validation of a high-quality composite real-world mortality endpoint. *Heath Serv Res*. 2018;53(6):4460-4476. doi: 10.1111/1475-6773.12872.

Hernan MA, Robins JM. Using big data to emulate a target trial when a randomized trial is not available. *Am J Epidemiol*. 2016;183(8):758-764. doi: 10.1093/aje/kwv254.

Klein JP, Moeschberger MM. *Survival analysis. Techniques for censored and truncated data*. 2^nd^ ed; 2003.

Pant S, Schuler M, Iyer G, et al. Erdafitinib in patients with advanced solid tumours with FGFR alterations (RAGNAR): an international, single-arm, phase 2 study. *Lancet Oncol*. 2023;24(8):925-935. doi: 10.1016/S1470-2045(23)00275-9.

Schoenfeld DA. Sample-size formula for the proportional-hazards regression model. *Biometrics*. 1983;39:499-503.

Yadav K, Lewis RJ. Immortal time bias in observational studies. *JAMA*. 2021;325(7):686-687. doi: 10.1001/jama.2020.9151.

Zhang Q, Gossai A, Monroe S, Nussbaum NC, Parrinello CM. Validation analysis of a composite real-world mortality endpoint for patients with cancer in the United States. *Health Serv Res*. 2021;56(6):1281-1287. doi: 10.1111/1475-6773.13669.

1. For patients with multiple FMI CGP assays performed, the first FMI CGP report date was used. [↑](#footnote-ref-2)
2. The “90-day rule” is intended to exclude patients who are likely to be missing treatment data, laboratory tests/results, vital readings, or clinical documents such as physician notes (eg, in the case patients were treated at a practice outside of the network before transferring treatment to a practice in network). [↑](#footnote-ref-3)
